# A Systematic Review of the Cost-Effectiveness of Biologics for the Treatment of Inflammatory Bowel Diseases

**DOI:** 10.1371/journal.pone.0145087

**Published:** 2015-12-16

**Authors:** Saara Huoponen, Marja Blom

**Affiliations:** Faculty of Pharmacy, University of Helsinki, Helsinki, Finland; Università degli Studi di Palermo, ITALY

## Abstract

**Background:**

Biologics are used for the treatment of inflammatory bowel diseases, Crohn´s disease and ulcerative colitis refractory to conventional treatment. In order to allocate healthcare spending efficiently, costly biologics for inflammatory bowel diseases are an important target for cost-effectiveness analyses. The aim of this study was to systemically review all published literature on the cost-effectiveness of biologics for inflammatory bowel diseases and to evaluate the methodological quality of cost-effectiveness analyses.

**Methods:**

A literature search was performed using Medline (Ovid), Cochrane Library, and SCOPUS. All cost-utility analyses comparing biologics with conventional medical treatment, another biologic treatment, placebo, or surgery for the treatment of inflammatory bowel diseases in adults were included in this review. All costs were converted to the 2014 euro. The methodological quality of the included studies was assessed by Drummond’s, Philips’, and the Consolidated Health Economic Evaluation Reporting Standards checklist.

**Results:**

Altogether, 25 studies were included in the review. Among the patients refractory to conventional medical treatment, the incremental cost-effectiveness ratio ranged from dominance to 549,335 €/Quality-Adjusted Life Year compared to the incremental cost-effectiveness ratio associated with conventional medical treatment. When comparing biologics with another biologic treatment, the incremental cost-effectiveness ratio ranged from dominance to 24,012,483 €/Quality-Adjusted Life Year. A study including both direct and indirect costs produced more favorable incremental cost-effectiveness ratios than those produced by studies including only direct costs.

**Conclusions:**

With a threshold of 35,000 €/Quality-Adjusted Life Year, biologics seem to be cost-effective for the induction treatment of active and severe inflammatory bowel disease. Between biologics, the cost-effectiveness remains unclear.

## Introduction

Crohn’s disease (CD) and ulcerative colitis (UC) are the principal types of inflammatory bowel diseases (IBDs) [1,2]. IBDs, which are chronic diseases, are characterized by inflammation of the mucosal lining of the gastrointestinal tract. Their worldwide incidence has increased during the last decade, but the annual incidence and prevalence of CD and UC are the highest in Northern Europe and in North America [3]. The incidence of CD is 12.7 per 100,000 person-years in Europe and 20.2 person-years in North America, while the incidence of UC is 24.3 in Europe and 19.2 in North America. Unemployment, sick leave, and permanent work disability are more commonly associated with patients with IBD than with the general population [4]. IBDs affect mainly young adults, causing an even greater economic burden.

Treatment of IBDs is aimed at relieving the symptoms and complications of IBDs as well as preventing recurrence and improving the patient’s quality of life [5,6]. IBD patients usually require lifelong medical treatment. Both CD and UC are treated with conventional medical treatment comprising corticosteroids, aminosalicylates, and immunomodulators (e.g., azathioprine, 6-mercaptopurine, and methotrexate). Other treatment options include surgery and diet therapy [5–7]. Biologic drugs based on two different mechanisms of action are currently available for the treatment of IBDs [8]. Infliximab (IFX), adalimumab (ADA), golimumab, and certolizumab pegol (CTZ) are tumor necrosis factor (TNF) inhibitors while natalizumab (NTZ) and vedolizumab target the α4-integrin [9,10]. Biologics are used to treat IBD refractory to corticosteroids or immunomodulators or IBD patients who are steroid-dependent or steroid-intolerant [5,6]. However, biologics are significantly more expensive than conventional drugs [11,12]. The introduction of TNF inhibitors has changed the cost profile of healthcare costs of IBDs [12,13]. Nowadays the main source of costs is drugs, especially TNF inhibitors, while earlier the healthcare costs were mainly driven by the hospitalization and the surgery. Biologics have been shown to be effective in inducing and maintaining remission of IBD [5,6,8,14–16]. Despite their proven efficacy, treatment failures may manifest as primary non-response, secondary loss of response, or failure of re-induction therapy [5,6,17]. Patients who fail to respond to TNF inhibitor may benefit from biologic drug with a different mechanism of action, while an alternative TNF inhibitor may be an effective treatment strategy in case of loss of response over time. The evidence on the cost-effectiveness of biologics for the treatment of IBD is limited, and the results of previous systematic reviews are inconsistent and incomplete [18–22].

The field of cost-effectiveness analysis (CEA) involves the comparison of health interventions based on both costs and effectiveness [23,24]. Cost-utility analysis (CUA) is a type of CEA. The outcome measure of the CUA is the incremental cost-effectiveness ratio (ICER), representing the difference in costs between two alternatives divided by the difference in effectiveness between the same two alternatives. An intervention dominates another if its effectiveness is higher and its costs are lower [24]. While the health effects are measured in natural units in the CEA, the measure of consequences in the CUA is the Quality-Adjusted Life Year (QALY) [24,25]. The QALY takes into account both the quantity and quality of life and can be measured by either direct or indirect methods. Costs are classified as direct and indirect costs [24,26]. Direct costs denote the resources consumed, while indirect costs are costs due to the loss of productivity related to illness or death. CEA can be conducted using an empirical, observational, or modeling approach [24]. The modeling study appears to be the most common type of CEA, combining clinical data and cost data from many sources. Modeling studies can be tested by sensitivity analysis.

The CEAs provide valuable information for health care decision-makers and enable efficient spending [24]. The aim of this systematic review is to evaluate existing relevant evidence regarding the cost-effectiveness of biologics for the treatment of IBDs. The cost-effectiveness of biologics is compared with placebo treatment, conventional medical treatment, surgery, and another biologic treatment for adults with diagnosed IBD. The aim of this review is also to analyze the source of effectiveness of CEAs. Furthermore, this review assesses the quality of the included CEAs using three different quality assessment checklists.

## Materials and Methods

### Literature Search

A comprehensive literature search on the cost-effectiveness of biologics for the treatment of IBDs was performed using Medline (Ovid), Cochrane Library (Cochrane Database of Systematic Reviews, Database of Abstracts of Reviews of Effects, Cochrane Central Register of Controlled Trials, Cochrane Methodology Register, Heath Technology Assessment Database, and NHS Economic Evaluation Database), and SCOPUS (including Embase) in June 2014. The search strategies were developed together with an information specialist. The reference lists of relevant articles were scrutinized. Furthermore, the grey literature and other relevant websites and databases (Centre for Reviews and Dissemination, Current Controlled Trials, Clinical Trials.gov, and PROSPERO) were hand-searched for relevant studies.

The electronic search strategy was based on patients (IBD, CD, or UC), intervention (biologics), and outcomes (ICER) in different spellings (S1 File). The biologics granted a marketing authorization by the European Medicines Agency (EMA) or US Food and Drug Administration (FDA) before May 2014 were included in the literature search strategy [9,10]. No restriction was set based on the year of the publication.

### Study Selection

The study selection was based on the inclusion and the exclusion criteria formulated by the framework of PICOTS i.e., population, intervention, comparator, outcome, timing, and setting (S1 Table) [27]. The study selection procedure encompassed three main stages. At the first stage, hits from the electronic databases were imported into reference management software (RefWorks). After removing duplicate citations, the second stage focused on the evaluation of the remaining studies based on their titles and abstracts. Studies clearly indicated as irrelevant to the study subject were excluded. The full articles retrieved that met the inclusion criteria are included in the current review. The identified abstracts and full texts were screened for eligibility by one reviewer (SH) and the second reviewer (MB) was consulted.

### Data Extraction

Our data extraction form was based on the Cochrane Handbook for Systematic Reviews of Intervention and the abstract form of the NHS Economic Evaluation Database [28,29]. The following items were extracted: patients, interventions, controls, study design (the type of economic evaluation and modeling, perspective, time horizon, country, included costs, the methods of measuring and valuing outcomes and benefits, discount rate, currency, price year, and the type of sensitivity analysis) and outcomes (total costs and benefits, ICER, and the results of sensitivity analysis). In order to facilitate the comparison of estimates collected from different studies, all costs were converted to 2014 euro using the exchange rates of the European Central Bank and the value of money index published by Statistics Finland [30,31]. Data were extracted using Microsoft Excel and performed by one assessor (SH) and ambiguities were solved by another assessor (MB) for accuracy.

### Quality Assessment

The methodological quality of the studies was assessed using three standardized checklists. All studies were assessed using Drummond’s checklist, published by the British Medical Journal Working Party, and the Consolidated Health Economic Evaluation Reporting Standards (CHEERS) guidelines [32,33]. In addition, economic evaluations using modeling methods were assessed using Philips’ checklist [34]. The quality assessment was conducted by one assessor (SH) and ambiguities were resolved by consulting another assessor (MB).

### Synthesizing Data

The results of the included CUAs were stratified into 4 subgroups by the type of previous treatments: 1) the cost-effectiveness of biologics in patients without previous treatment, 2) the cost-effectiveness of biologics in patients with previous conventional medical treatment, 3) the cost-effectiveness of biologics in patients with previous surgery, and 4) the cost-effectiveness of biologics in patients with previous biologic treatment. Biologic treatments were stratified under three dosing regimens: a single dose, an episodic treatment, or a maintenance treatment. ICERs were presented as principal outcomes. In this study, we analyzed the cost-effectiveness of biologics using the willingness-to-pay threshold of 35,000 €/QALY. A quantitative synthesis of the study results was not possible because of the heterogeneity in participants, interventions and study designs.

## Results

### Literature Search

**Fig 1:**
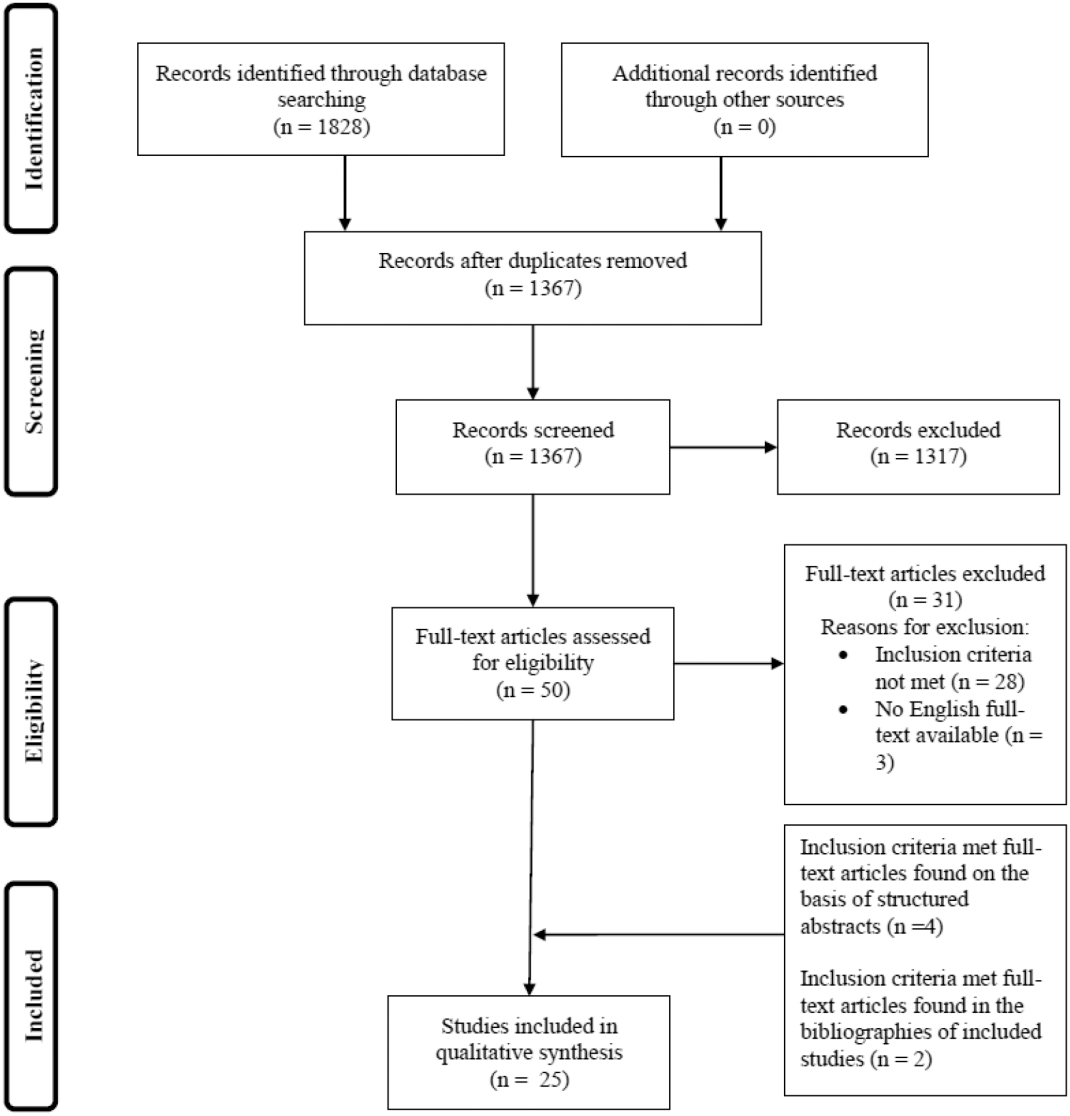
Flow chart of study selection.

The database search identified 1828 references, of which 461 were removed as duplicates, leaving 50 studies to be screened by abstracts and titles for further evaluation. After the assessment of the full text, 31 studies were excluded (S2 File) and 19 studies were included in the review. Additionally, six full-text articles were included, of which two were found from the bibliographies of already included studies [35,36] and four from the structured abstracts identified by the literature search [19,22,37,38]. The hand search revealed no further publications. Altogether, 25 studies were included in the review [19,21,22,35–56]. Study selection is presented in a flow diagram in Fig 1.

### Characteristics of Studies Included in the Review

All CUAs involved economic evaluation modeling, of which 17 and 7 were focused on CD and UC, respectively, while one study featured both diagnoses. IFX, ADA, NTZ, and CTZ were studied in 22, 8, two, and one CUAs, respectively. All studies were conducted in North America or in Europe. One study considered both direct and indirect costs [49]. Three studies were modelled for lifetime [43,44,46], while most studies used one year time horizon. The study designs, the interventions, and the comparators of the CUAs were heterogeneous. Table 1 presents the characteristics of the studies.

**Table 1:** Characteristics of the studies.

Author, Year of publication, Country	Patients	Biologic treatment	Comparative treatment	Perspective	Time horizon, Type of modelling	Source of effectiveness	Source of utility data, Instruments or valuation methods for utility measures	Discount rate
**Crohn’s disease**								
Ananthakrishnan et al. 2011, USA [39]	CD patients, who were in surgical remission after their first ileocecal resection	Upfront IFX or Tailored IFX	Antibiotic	Third-party payer	1 year, Decision tree model	Meta-analysis systematic review, cohort studies	Utility values derived from study by Casellas et al [57], for surgery and after surgery by a panel of UK gastroenterologists, EQ-5D, utilities valued using UK tariffs	-
Ananthakrishnan et al. 2012, USA [40]	Moderate-to severe luminal CD, loss of response to two prior TNF inhibitors	NTZ	CTZ	Third-party payer	1 year, Decision tree model	RCTs, multi-center report, cohort study	Utility values derived from study by Gregor et al [58], SG, utility scores classified by CDAI	-
Arseneau et al. 2001, USA [41]	Fistulizing CD	First-line IFX, second-line 6MP+MET or IFX episodic reinfusion or First-line 6MP+MET, second-line IFX episodic reinfusion	6MP+MET	Third-party payer	1 year, Markov model	Systematic review	Preference weights were directly elicited from CD patients and healthy individuals, SG	3% for costs and benefits
Assasi et al. 2009, Canada [22]	Moderate-to severe CD (CDAI ≥ 200), refractory to conventional medical treatment	IFX 5 mg/kg induction and maintenance treatment or ADA induction treatment (160 mg at week 0, 80 mg at week 2) and maintenance treatment (40 mg)	Conventional medical treatment or ADA induction treatment (160 mg at week 0, 80 mg at week 2) and maintenance treatment (40 mg)	Third-party payer	5 years, Markov model	Systematic review	Utility values derived from study by Gregor et al [58], SG	5% for costs and QALYs
Blackhouse et al. 2012, Canada [42]	Refractory to conventional medical treatment (CDAI > 200)	IFX 5 mg/kg induction and maintenance treatment or ADA induction treatment (160 mg at week 0, 80 mg at week 2) and maintenance treatment (40 mg)	Conventional medical treatment or ADA induction treatment (160 mg at week 0, 80 mg at week 2) and maintenance treatment (40 mg)	Third-party payer	5 years, Markov model	Systematic review	Utility values derived from study by Gregor et al [58], SG	5% for costs and QALYs
Bodger et al. 2009, UK [43]	Moderate-to severe active CD, (CDAI > 220)	IFX 5 mg/kg + conventional medical treatment or ADA 80 mg at week 0, 40 mg at week 2, 40 mg for maintenance + conventional treatment	Conventional treatment	Payer, UK NHS	60 years (lifetime), duration of treatment 1 or 2 years, Markov model	Systematic review	EQ-5D converted from CDAI (EQ-5D = 0.9168–0.0012 × CDAI, algorithm by Buxton et al [59])	3.5% for costs and QALYs
Clark et al. 2003, UK [44]	a, b) Severe active CD, c) Fistulizing CD	IFX 5 mg/kg single dose or IFX 5 mg/kg episodic re-treatment if lost response or IFX 5 mg/kg maintenance treatment	Placebo	Unclear	a) Lifetime, b) Unclear, probably 1 year, c) 1 year, a) Markov model, b,c) Type of modeling unclear	a, b) RCTs, c)RCT	a, b) Utility values derived from study by Gregor et al [58], SG, utility scores corresponding to the exact CDAI states, c) Combination of CDAI and PDAI score into utility using an unpublished formulae	6% for costs and 1.5% for QALYs
Doherty et al. 2012, USA [45]	CD patients undergone intestinal resection	IFX 5 mg/kg induction and maintenance treatment	AZA / 6MP	Societal	1 year, 5 years, Decision analysis model	Meta-analysis	Utility values derived from study by Gregor et al [58], SG, utility scores classified by CDAI	3%
Dretzke et al. 2011, UK [21]	Moderate-to-severe CD, refractory to conventional medical treatment	IFX induction treatment or IFX maintenance treatment or ADA induction treatment or ADA maintenance treatment	Conventional medical treatment or IFX induction treatment or ADA induction treatment	Payer, UK NHS	1 year, Markov model	Systematic review	Utility values derived from study by Gregor et al [58], assumptions for surgery, TTO, EQ-5D	3.5% for costs and QALYs
Jaisson-Hot et al. 2004, France [46]	Moderate-to-severe active ileocolonic non fistulizing CD (CDAI 220–440), resistant to conventional medical treatment	IFX with retreatment when patients relapse/do not respond or IFX maintenance treatment	Surgery involving conventional medical treatment	Third-party payer	Lifetime, Markov model	RCT, expert opinion, cohort study	Utility values derived from study by Gregor et al [58], SG, utility scores classified by CDAI	5% for costs and QALYs
Kaplan et al. 2007, USA [47]	CD patients, no response to 5 mg/kg of IFX	IFX dose escalation to 10 mg/kg	ADA initiation	Unclear	1 year, Decision analysis model	RCTs, cohort study	Utility values derived from study by Gregor et al [58], SG, utility scores classified by CDAI	-
Lindsay et al. 2008, UK [48]	Active luminal non-fistulizing CD (CDAI 220–400) or Active fistulizing CD	IFX 5 mg/kg	Conventional medical treatment	Payer, UK NHS	5 years, Markov model	RCTs, cohort study	Utility values derived from study by Casellas et al [57], for surgery and after surgery by a panel of UK gastroenterologists, EQ-5D, utilities valued using UK tariffs	3.5% for costs and QALYs
Loftus et al. 2009, UK [49]	Moderate-to-severe CD	ADA	Conventional medical treatment	Payer, UK NHS	1 year, Type of modeling unclear	RCTs	Utility values derived from study by Gregor et al [58], SG, utility scores classified by CDAI	3.5% for costs and QALYs
Marchetti et al. 2013, Italy [50]	Newly diagnosed luminal moderate-to-severe CD patients	Top-down strategy: IFX 5 mg/kg+AZA à additional IFX 5 mg/kg+AZA à MPR+AZA	Step-up strategy: MPR à MPR+AZA à IFX+AZA	Third-party payer	5 years, Markov model	RCT, cohort studies	EQ-5D and SF-6D converted from CDAI, SF-6D = 0.8129–0.00076 × CDAI, EQ-5D = 0.9168–0.0012 × CDAI, by Buxton et al	3.5% for costs and QALYs
Marshall et al. 2002, Canada [19]	CD patients resistant to conventional medical treatment	IFX 5 mg/kg single dose, relapses treated with conventional treatment or IFX 5 mg/kg single dose, relapses treated with IFX 5 mg/kg single dose or IFX 5 mg/kg single dose with responding patients IFX 5 mg/kg maintenance treatment, relapses treated with conventional medical treatment	Conventional treatment or IFX 5 mg/kg single dose, relapses treated with conventional treatment or IFX 5 mg/kg single dose, relapses treated with IFX 5 mg/kg single dose	Third-party payer	1 year, Markov model	RCTs, cohort study	Utility values derived from study by Gregor et al [58], SG, utility scores classified by CDAI	-
Saito et al. 2013, UK [51]	Biologic-naive CD patients refractory to conventional medical treatment (CDAI 220–450)	IFX 5 mg/kg+AZA	IFX 5 mg/kg	Payer, UK NHS	1 year, Decision tree model	RCTs, observational study	Utility values derived from study by Gregor et al [58], expert opinion data for non-responding active disease, SG, utility scores classified by CDAI	-
Tang et al. 2012, USA [52]	Moderate-to-severe CD (CDAI 220–450), refractory to conventional medical treatment and naïve to biologics	ADA or CTZ or NTZ	IFX	Third-party payer	1 year, Decision analytic model	RCTs	Utility values derived from study by Gregor et al [58], SG, utility scores classified by CDAI	-
Yu et al. 2009, USA [56]	Active moderate-to-severe CD, candidate for anti-TNF maintenance treatment	ADA (40 mg every other week) maintenance treatment	IFX 5 mg/kg maintenance treatment	Third-party payer	1 year, Type of modeling unclear	RCTs	Utility values derived from study by Gregor et al [58], SG, utility scores classified by CDAI	-
**Ulcerative colitis**								
Assasi et al. 2009, Canada [22]	Moderate-to-severe UC, refractory to conventional medical treatment	IFX 5 mg/kg followed by switching to ADA 160 mg when relapse or IFX 5 mg/kg followed by IFX 10 mg/kg dose escalation when relapse	Conventional medical treatment or IFX 5 mg/kg followed by switching to ADA 160 mg when relapse	Third-party payer	5 years, Markov model	Systematic literature review	TTO, Utility weights elicited from UC patients	5% for costs and QALYs
Bryan et al. 2008, UK [37]	Acute exacerbation of UC that require hospitalization, inadequate response to conventional medical treatment	IFX 5 mg/kg+IV CST	Placebo or CYC or Surgery	Payer, UK NHS	1 year, Decision analytic model	RCTs	EQ-5D, Utility weights derived from UC patients	3.5% for costs and QALYs
Chaudhary et al. 2013, Netherlands [36]	Severely active UC, hospitalized with an acute exacerbation of UC, refractory to IV CST	IFX 5 mg/kg	IV CYC or Surgery	Third-party payer	1 year, Decision analytic model, beyond the first year a Markov model	RCTs	EQ-5D, valued using UK tariffs, TTO for post-surgery complications, Utility scores classified by SCAI, Utility weights derived from UC patients	4% for costs, 1.5% for QALYs
Hyde et al. 2007, UK [38]	Moderate-to-severe active UC, an inadequate response to conventional medical treatment	IFX 5 mg/kg	Conventional treatment	Payer, UK NHS	10 years, Markov model	RCTs	EQ-5D, Utility weights derived from UC patients	3.5% for costs and QALYs
Punekar et al. 2010, UK [35]	Severely active UC, hospitalized with an acute exacerbation of UC, refractory to IV CST	IFX 5 mg/kg + IV CST	IV CST or CYC+IV CST or Surgery	Payer, UK NHS	1 year, Decision analytic model, beyond the first year a Markov model	A network meta-analysis	EQ-5D, valued using UK tariffs, TTO for post-surgery complications, Utility scores classified by SCAI, Utility weights derived from UC patients	3.5% for costs and QALYs
Tsai et al. 2008, UK [53]	Moderate-to-severe UC	Scheduled maintenance treatment with IFX 5 mg/kg	Conventional medical treatment	Payer, UK NHS	10 years, Markov model	RCTs	EQ-5D, valued using UK tariffs, TTO for post-surgery complications, Utility scores classified by SCAI, Utility weights derived from UC patients	3.5% for costs and QALYs
Ung et al. 2014, Canada [54]	Moderate or moderately severe UC, CST-dependent and refractory to thiopurine	IFX 5 mg/kg	Conventional medical treatment	Third-party payer	10 years, Markov model	RCTs, real life rates	TTO, VAS, Utility weights derived from UC patients	5% for costs and QALYs
Xie et al. 2009, Canada [55]	Moderate-to-severe UC, refractory to conventional medical treatment	IFX 5 mg/kg followed by IFX 10 mg/kg dose escalation when relapse or IFX 5 mg/kg followed by switching to ADA 160 mg when relapse	Conventional medical treatment	Third-party payer	5 years, Markov model	Fixed-effect meta-analysis	TTO, Utility weights derived from UC patients	5% for costs and QALYs

^→^, Transition because of the clinical worsening in the earlier state; 6MP, Mercaptopurine; ADA, Adalimumab; AZA, Azathioprine; CD, Crohn’s disease; CDAI, Crohn’s disease activity index; CST, Corticosteroid; CTZ, Certolizumab pegol; CYC, Cyclosporine; EQ-5D, European Quality of Life Instrument 5 D; IBDQ-36, Inflammatory Bowel Disease Questionnaire 36; IFX, Infliximab; IV, Intravenous; MET, Metronidazole; MPR, Methylprednisolone; NTZ, Natalizumab; PDAI, Pouchitis Activity Index; QALY, Quality-Adjusted Life Year; RCT, Randomized controlled trial; SCAI, Simple Clinical Colitis Activity Index; SF-6D, Short Form-6 dimension; SG, Standard gamble; TNF, Tumor necrosis factor; TTO, Time Trade-Off; UC, Ulcerative colitis; UK NHS, National Health Service (England); VAS, Visual Analog Scale.

### Cost-Effectiveness of Biologics in Patients with No Previous Treatment

In two studies, the cost-effectiveness of biologics was evaluated in CD patients with no previous treatment (Table 2) [41,50]. In comparison with conventional drugs for the treatment of fistulizing CD, ICERs ascended in excess of 400,000 €/QALY [41] while for newly diagnosed luminal CD IFX was dominant [50]. No CEAs of biologics in UC patients without earlier treatment were found (Table 3).

**Table 2:** Cost-effectiveness of biologics for the treatment of Crohn’s disease (CD).

Study	Intervention (Biologic treatment)	Comparison treatment	ICER^a^ €^d^/QALY (including only direct^b^ costs)	ICER^a^ €^d^/QALY (including both direct^b^ and indirect^c^ costs)	Results of deterministic sensitivity analysis (€^d^/QALY)	Source of research funding
**Cost-effectiveness of biologics in CD patients with no previous treatment**						
Arseneau et al. 2001 [41]	First-line IFX	6MP+MET	438,617	-	219,353–dominance by comparison treatment	NIDDK
	IFX episodic reinfusion	6MP+MET	445,477	-	127,314–dominance by comparison treatment	NIDDK
	Second-line IFX episodic reinfusion	6MP+MET	465,394	-	155,109–comparison treatment is cost-saving	NIDDK
Marchetti et al. 2013 [50]	Top-down: IFX	Step-up: IFX	Dominance by intervention treatment	-	Dominance by intervention treatment–93,401	None declared
**Cost-effectiveness of biologics in CD patients with earlier conventional medical treatment**						
Assasi et al. 2009 [22]	IFX	Conventional medical treatment	155,295	-	142,742–254,029	Canadian federal, provincial, and territorial governments
	ADA	Conventional medical treatment	134,643	-	120,307–474,352	Canadian federal, provincial, and territorial governments
	IFX	ADA	314,250	-	154,436–Dominance by comparison treatment	Canadian federal, provincial, and territorial governments
Blackhouse et al. 2012 [42]	IFX	Conventional medical treatment	164,626	-	74,434–344,212	Not stated, one of authors has received an honorarium from Abbott and acted as a consultant for Centocor Ortho Biotech Services
	ADA	Conventional medical treatment	142,733	-	63,679–297,508	Not stated, one of authors has received an honorarium from Abbott and acted as a consultant for Centocor Ortho Biotech Services
	IFX	ADA	331,132	-	157,253–Dominance by comparison treatment	Not stated, one of authors has received an honorarium from Abbott and acted as a consultant for Centocor Ortho Biotech Services
Bodger et al. 2009 [43]	IFX	Conventional treatment	31,982 (Duration of treatment 1 year); 35,759 (Duration of treatment 2 years)	-	31,227–Dominance by comparison treatment	The Welsh Office for Research and Development for Health and Social Care
	ADA	Conventional treatment	12,071 (Duration of treatment 1 year); 17,309 (Duration of treatment 2 years)	-	12,692–304,912	The Welsh Office for Research and Development for Health and Social Care
Clark et al. 2003 [44]	IFX single dose	Placebo	a) 11,725 b) 236,836 (scenario 1) 163,179 (scenario 2) c) 178,503–215,253	-	b) 236,836–529,754 (scenario 1); 163,179–373,921 (scenario 2) c) 143,502–215,253	NICE (UK)
	IFX episodic re-treatment if lost response	Placebo	a) 18,200 b) 126,459 (scenario 1); 108,530 (scenario 2)	-	a) 34,651–95,901 b) 82,197–126,459 (scenario 1); 70,544–108,530 (scenario 2)	NICE (UK)
	IFX maintenance treatment	Placebo	a) 147,702	-	-	NICE (UK)
Dreztke et al. 2011 [21]	IFX induction treatment	Conventional medical treatment	Dominance by intervention treatment (Severe CD); 162,941 (Moderate CD)	-	17,346–123,198	NICE (UK)
	IFX maintenance treatment	Conventional medical treatment	118,015 (Severe CD); 549,335 (Moderate CD)	-	63,127–2,764,027	NICE (UK)
	ADA induction treatment	Conventional medical treatment	Dominance by intervention treatment	-	Dominance by intervention treatment	NICE (UK)
	ADA maintenance treatment	Conventional medical treatment	13,387 (Severe CD); 276,539 (Moderate CD)	-	Dominance by intervention treatment–1,180,345	NICE (UK)
	IFX maintenance treatment	IFX induction treatment	8,689,409 (Severe CD); 24,012,483 (Moderate CD)	-	553,635–8,568,483	NICE (UK)
	ADA maintenance treatment	ADA induction treatment	8,603,033 (Severe CD); 24,012,483 (Moderate CD)	-	Dominance by intervention treatment–8,810,335	NICE (UK)
Jaisson-Hot et al. 2004 [46]	IFX re-treatment	Surgery	77,002	-	77,002–dominance by comparison treatment	Not stated
	IFX maintenance treatment	Surgery	947,769	-	947,769–dominance by comparison treatment	Not stated
Lindsay et al. 2008 [48]	IFX	Conventional medical treatment	45,137 (Severe active luminal CD); 51,397 (Fistulizing CD)	-	41,032–67,111 (Severe active luminal CD); 46,724–76,367 (Fistulizing CD)	Schering Plough Ltd
Loftus et al. 2009 [49]	ADA	Conventional medical treatment	27,751 (Severe CD); 58,271 (Moderate-to-severe CD)	9,069 (Severe CD); 42,554 (Moderate-to-severe CD)	11,315–59,133 (Severe CD); 30,876–99,455 (Moderate-to-severe CD)	Abbott Laboratories
Marshall et al. 2002 [19]	IFX single dose	Conventional treatment	162,181	-	34,908–Dominance by comparison treatment	CCOHTA (now CADTH)
	IFX single dose with re-treatment	IFX single dose	429,715	-	Dominance by intervention treatment–533,605	CCOHTA (now CADTH)
	IFX maintenance treatment	IFX single dose with re-treatment	623,013	-	1,620–736,716	CCOHTA (now CADTH)
Saito et al. 2013 [51]	IFX+AZA	IFX	34,549	-	23,776–63,178	CISA
Tang et al. 2012 [52]	ADA	IFX	-	-	-	Not stated
	CTZ	IFX	-	-	-	Not stated
	NTZ	IFX	-	-	-	Not stated
Yu et al. 2009 [56]	ADA maintenance treatment	IFX maintenance treatment	Dominance by intervention treatment	-	Dominance by intervention treatment	Abbott Laboratories
**Cost-effectiveness of biologics in CD patients with earlier surgical treatment**						
Ananthakrishnan et al. 2011 [39]	Upfront IFX	Antibiotic	2,268,986	-	594,301–5,485,175	None declared, one author receives research support from Procter and Gamble and Warner Chilcott
	Tailored IFX	Antibiotic	Dominance by comparison treatment		Dominancy by comparison treatment	None declared, one author receives research support from Procter and Gamble and Warner Chilcott
Doherty et al. 2012 [45]	IFX	AZA/6MP	1,449,979 (Time horizon 1 year); 1,823,102 (Time horizon 5 years)	-	1,449,979–Dominance by comparison treatment	Pfizer Inc. and Merck & Co. One of authors receives research funding from Proctor & Gamble, Shire and Salix
**Cost-effectiveness of biologics in CD patients with earlier biological treatment**						
Ananthakrishnan et al. 2012 [40]	NTZ	CTZ	314,020	-	Dominance by intervention treatment—Dominance by comparison treatment	None declared
Kaplan et al. 2007 [47]	IFX dose escalation	ADA	311,432	-	46,862–Dominance by comparison treatment	None declared, authors have received research grants from UCB Pharma, Abbott Laboratories, Centocor, Bristol-Myers Squibb, Elan Pharmaceuticals, Prometheus Laboratories, Otsuka America Pharmaceuticals Inc

ADA, Adalimumab; CEGIIR, Centre of Excellence for Gastrointestinal Inflammation and Immunity Research; CST, Corticosteroid; CYC, Cyclosporine; IFX, Infliximab; IV, Intravenous; NICE, National Institute for Health and Care Excellence; RCT, Randomized controlled trial; QALY, Quality-Adjusted Life Year; TTO, Time Trade-off; VAS, Visual analog scale.

^a^The difference in costs divided by the difference in health effects.

^b^The resources consumed.

^c^Productivity costs for the patient and family members.

^d^All costs converted into 2014 euro.

**Table 3:** Cost-effectiveness of biologics for the treatment of ulcerative colitis (UC).

Study	Intervention (Biologic treatment)	Comparison treatment	ICER^a^ €^d^/QALY (including only direct^b^ costs)	ICER^a^ €^d^/QALY (including both direct^b^ and indirect^c^ costs)	Results of deterministic sensitivity analysis (€^d^/QALY)	Source of research funding, Conflict of interest of authors
**Cost-effectiveness of biologics in UC patients with earlier conventional medical treatment**						
Assasi et al. 2009 [22]	IFX followed by IFX dose escalation when relapse	Conventional medical treatment	407,499	-	294,007–629,598	Canadian federal, provincial, and territorial governments
	IFX followed by switching to ADA when relapse	Conventional medical treatment	253,537	-	191,701–373,298	Canadian federal, provincial, and territorial governments
	IFX followed by IFX dose escalation when relapse	IFX followed by switching to ADA when relapse	Dominance by comparison treatment	-	-	Canadian federal, provincial, and territorial governments
Bryan et al. 2008 [37]	IFX	CYC	33,486	-	2,399–108,262	NICE (UK)
	IFX	Placebo	20,829		7,745–24,268	NICE (UK)
	IFX	Surgery	24,293	-	2,470–109,612	NICE (UK)
Chaudhary et al. 2013 [36]	IFX	IV CYC	26,479	-	17,609–38,985	Merck & Co
	IFX	Surgery	15,967	-	11,614–24,475	Merck & Co
Hyde et al. 2007 [38]	IFX	Conventional treatment	72,711	-	29,363–101,989	NICE (UK)
Punekar et al. 2010 [35]	IFX	IV CST	19,198	-	Dominance by intervention treatment–94,322	Schering-Plough Ltd
	IFX	CYC+IV CST	30,871	-	Dominance by intervention treatment–108,272	Schering-Plough Ltd
	IFX	Surgery	22,161	-	Dominance by intervention treatment–109,279	Schering-Plough Ltd
Tsai et al. 2008 [53]	IFX maintenance treatment	Conventional medical treatment	46,041 (responders only); 33,067 (remission only)	-	353,367–144,921 (responders only); 24,726–78,511 (remission only)	Schering-Plough Ltd
Ung et al. 2014 [54]	IFX	Conventional medical treatment	Source of effectiveness based on RCTs: 115,639 (TTO); 99,663 (VAS), Source of effectiveness based on real-life studies: 66,949 (TTO); 60,101 (VAS)	-	Source of effectiveness based on RCTs: 54,777–248,016, Source of effectiveness based on real-life studies: 31,192–94,337	CEGIIR and the Alberta Innovates—Health Solutions supported Alberta IBD Consortium
Xie et al. 2009 [55]	IFX dose escalating when relapse	Conventional medical treatment	407,499	-	303,515–629,598	Not stated, Conflict of interest: Eli Lilly Canada Inc, GlaxoSmithKline Inc, Abbott Laboratories Ltd, Janssen-Ortho Inc., Hoffman-La Roche Ltd
	IFX switching to ADA when relapse	Conventional medical treatment	253,537	-	193,349–373,298	Not stated, Conflict of interest: Eli Lilly Canada Inc, GlaxoSmithKline Inc, Abbott Laboratories Ltd, Janssen-Ortho Inc., Hoffman-La Roche Ltd

ADA, Adalimumab; CEGIIR, Centre of Excellence for Gastrointestinal Inflammation and Immunity Research; CST, Corticosteroid; CYC, Cyclosporine; IFX, Infliximab; IV, Intravenous; NICE, National Institute for Health and Care Excellence; RCT, Randomized controlled trial; QALY, Quality-Adjusted Life Year; TTO, Time Trade-off; VAS, Visual analog scale.

^a^The difference in costs divided by the difference in health effects.

^b^The resources consumed.

^c^Productivity costs for the patient and family members.

^d^All costs converted into 2014 euro.

### Cost-Effectiveness of Biologics in Patients with Previous Conventional Medical Treatment

The cost-effectiveness of biologics in CD patients with previous conventional medical treatment was investigated in 12 studies (Table 2) [19,21,22,42–44,46,48,49,51,52,56]. For CD, ICERs for the biologics ranged from dominance to 549,335 €/QALY when compared with those of conventional medical treatment [19,21,22,42,43,48,49]. ADA as an intervention treatment resulted in more frequently lower ICERs than did IFX in comparison with conventional medical treatment [21,22,42,43]. IFX in comparison with surgery was not found to be cost-effective, with ICERs in excess of 77,000 €/QALY [46]. Between biologics cost-effectiveness was investigated in four studies [22,42,52,56]. ICERs above 300,000 €/QALY were seen when comparing IFX with ADA [22,42], while ADA maintenance treatment appeared to be dominant in comparison with IFX maintenance treatment [56].

Two studies evaluated the cost-effectiveness of biologics for different activity levels of CD resulting in more favorable ICERs for severe CD than for moderate CD [21,49]. The cost-effectiveness of biologics for fistulizing CD was examined in two studies (ICERs above 51,000 €/QALY) [44,48] and for luminal CD in two studies (ICERs above 45,000 €/QALY) [46,48]. Biologic induction treatment resulted in lower ICERs than maintenance treatment [21]. In one study, IFX and corticosteroid combination treatment was shown to be cost-effective in comparison with IFX monotherapy [51]. One study found more favorable ICER when including both direct and indirect costs than only direct costs [49]. The ICERs of the studies using lifetime horizon ranged from 11,725 to 947,769 €/QALY [43,44,46].

Eight CUAs evaluated the cost-effectiveness of biologics in UC patients with previous conventional medical treatment (Table 3) [22,35–38,53–55]. ICER remained below 35,000 €/QALY when comparing IFX with either conventional medical treatment, surgery, or placebo treatment for UC patients with acute exacerbation requiring hospitalization [35–37]. When investigating the cost-effectiveness of IFX for patients with moderate-to-severe UC, ICER ranged from 33,067 €/QALY to 407,499 €/QALY [22,38,53–55].

### Cost-Effectiveness of Biologics in Patients with Previous Surgery

The cost-effectiveness of biologics in CD patients having undergone intestinal resection was investigated in two CUAs (Table 2) [39,45]. IFX in comparison with conventional medical treatment was not cost-effective, producing extremely unfavorable ICERs above 1,400,000 €/QALY. No studies investigated the cost-effectiveness of biologics in UC patients with previous surgery (Table 3).

### Cost-Effectiveness of Biologics in Patients with Previous Biologic Treatment

The cost-effectiveness of biologics in CD patients with prior biologic treatment was investigated in two CUAs (Table 2) [40,47]. Neither IFX dose escalation in comparison with second-line ADA nor third-line CTZ in comparison with NTZ was cost-effective (ICERs above 300,000 €/QALY). No studies evaluated the cost-effectiveness of biologics in UC patients with prior TNF inhibitor treatment (Table 3).

### Effectiveness Data

In all studies, the source of effectiveness was based on at least one randomized controlled trial (RCT). One study used real life data published by specialized inflammatory bowel disease clinics and compared those findings with data from RCTs [54].

In 13 studies focused on CD, utility values were obtained by the Standard Gamble (SG) valuation technique [19,22,40–42,44–47,49,51,52,56]. In twelve studies, the utilities were derived from study by Gregor et al [58] which used the SG method in CD patients to define utility scores and correlated them with the Crohn’s disease Activity Index (CDAI) [19,22,40,42,44–47,49,51,52,56]. In two studies [39,48], health state preferences were driven from the study by Casellas et al [57] which estimated health state preferences of Spanish CD patients using the European Quality of Life Instrument 5 D (EQ-5D) and converted to utilities using UK tariffs. In two studies [43,50] the estimated EQ-5D utility scores were converted from CDAI scores based on the algorithm developed by Buxton et al [59].

In three studies concerning UC [35,36,53], the utility scores were obtained from an UC patient survey carried out in Cardiff Hospital using the EQ-5D and valued using UK tariffs [60]. Utilities were further classified into health states by a Simple Clinical Colitis Activity Index (SCAI). Two studies [22,55] used utilities from patients using Time Trade-off (TTO) valuation technique [61].

### Quality Assessment

**Fig 2:**
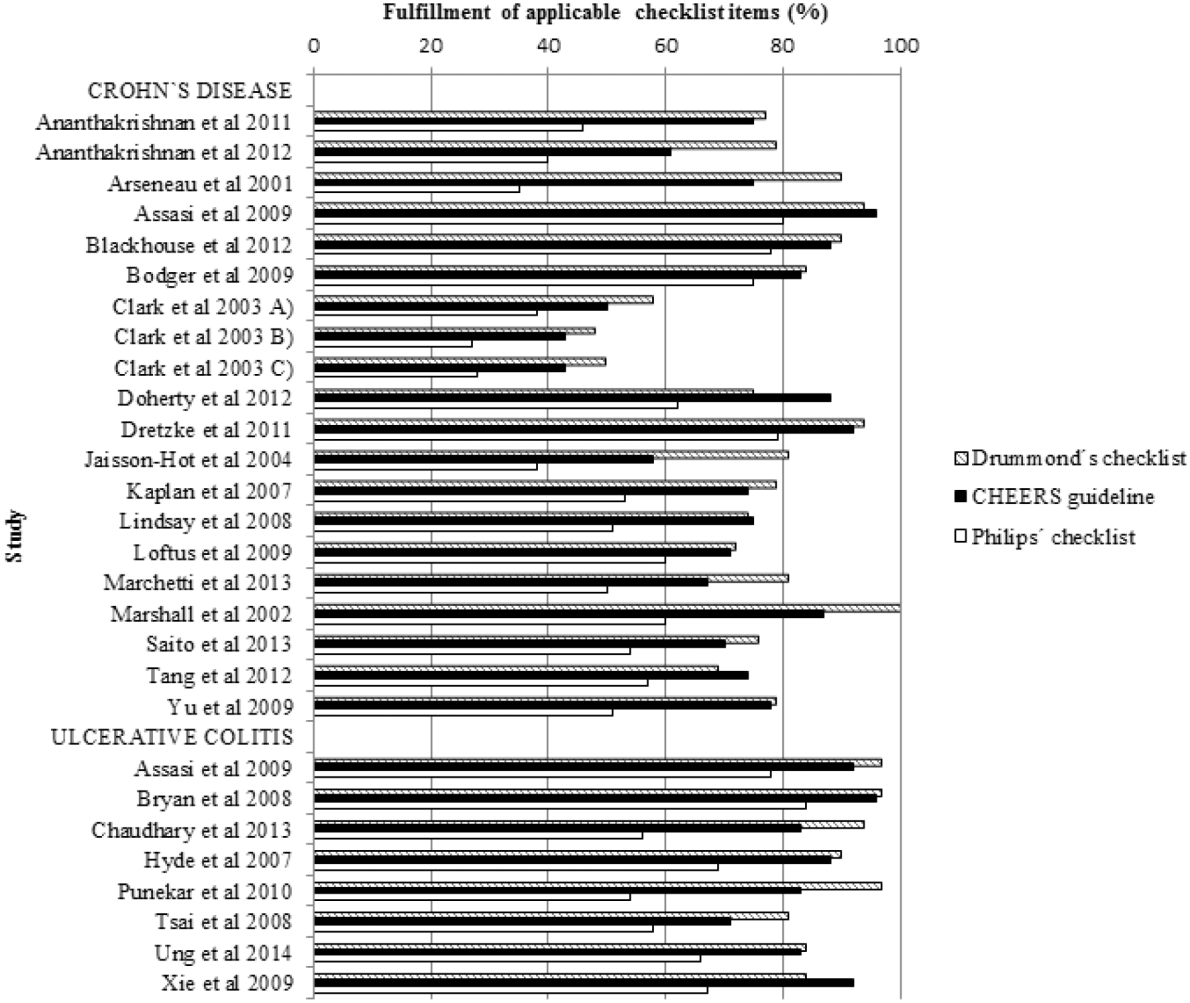
Quality assessment of the included studies.

The results of the quality assessment are shown in Fig 2. The mean amount of fulfilled criteria were 24.9 out of 35 (median 26, range 14–30), 29.6 out of 57 (median 29, range 14–46), and 18.2 out of 24 (median 18, range 10–23) for Drummond’s checklist, Philip’s checklist, and the CHEERS guideline, respectively. Studies by Assasi et al, Bryan et al, and Dretzke et al, which all are Health Technology Assessment (HTA) reports, fulfilled most criteria of the applicable items [21,22,37]. The quality elements most commonly omitted from the economic analyses were information on adjustments for data identification, baseline data, treatment effects, data incorporation, and assessment of uncertainty (S2 Table).

## Discussion

Altogether, 25 studies were included in this systematic review. The number of the included studies in this review was higher than in previously published reviews for IBD [18–22]. However, it is noteworthy that articles by Blackhouse et al and Xie et al are part of the study by Assasi et al [22,42,55]. A majority of the included studies used IFX or ADA as an intervention treatment, while NTZ and CTZ were investigated only in few studies, and none of the studies considered golimumab. Because of the variability in data input and heterogeneous study designs, the quantitative synthesis of the studies was not possible.

On the basis of the current review and willingness-to-pay threshold of 35,000 €/QALY, biologics in comparison with conventional medical treatment and placebo treatment were found to be cost-effective for severe CD in remission induction, while for maintenance treatment cost-effectiveness remained unclear. Biologics were not cost-effective in comparison with surgery for the treatment of severe CD. In moderate CD, biologics did not seem to be cost-effective. Biologics were found not to be cost-effective among CD patients having undergone intestinal resection. ADA was shown to be a more cost-effective biologic treatment option than IFX. Cost-effectiveness between individual biologics remained unclear, however.

Biologics were cost-effective for the treatment of acute exacerbation of severely active UC when compared with either conventional medical treatment, surgery, or placebo treatment. For moderate UC, biologics were not cost-effective. The cost-effectiveness between different biologics remained unclear in UC.

The literature search found five earlier published systematic reviews of the cost-effectiveness of biologics for IBDs [18–22]. In previous reviews, the conclusions have been contradictory and partially unreliable due to a low amount of included CUAs. Four out of five previous reviews evaluated the cost-effectiveness of biologics for CD [18–21], while one assessed the cost-effectiveness of biologics in both CD and UC [22]. IFX was the only biologic treatment in four reviews [19–22]. Meanwhile, the latest systematic review by Tang et al included IFX, ADA, CTZ, and NTZ and came to a conclusion that the biologics are cost-effective for CD in certain clinical situations which was congruent with this review [18]. The earlier review by Assasi et al [22] included one CUA [53] showing that scheduled maintenance treatment with IFX is a cost-effective option for UC [22]. The studies included in our review focused mainly on induction treatment and revealed no further evidence of the cost-effectiveness of biologic maintenance treatment for UC.

An important issue affecting the conclusions of CEAs relates to the established willingness-to-pay threshold. In the UK, the National Institute of Health and Clinical Excellence (NICE) supports treatments with ICER no higher than 30,000 £ (~35,000 €) per QALY [62], which we used as a threshold in this study. However, there has been much debate as to whether this threshold is too low, and many health care systems have not set a cost-effectiveness threshold at all [54]. The willingness-to-pay threshold commonly used by the Canadian Drug Expert Committee is 80,000 CDN$ (~75,000 €) per QALY [63], while the threshold of 50,000–100,000 US$ (~38,00–75,000 €) per QALY is often used in the US [64,65]. According to the World Health Organization, an intervention is cost-effective if the cost of intervention per QALY is less than three times the country’s annual gross domestic product [66]. Even if those thresholds had been used in this review, biologics would not have been deemed cost-effective in most studies. It should be noted that the selection of the willingness-to-pay threshold depends on the relevant context, including the disease burden and the budget of the setting in question.

Most studies used the perspective of the local public health care service or the insurance system while only paying attention to direct costs. Only one study included both direct and indirect costs and reported more favorable ICER when considering both direct and indirect costs in comparison with only direct costs [49]. No clear guidelines exist on how productivity losses should be determined causing concern for the validity of the cost estimates. Included cost components and their valuing methods can be difficult to identify based on the publications. Furthermore, productivity costs included in CUA may cause a risk of double-counting as the impact of morbidity is already included in the calculation of QALY [26]. The patient´s earnings and leisure activities affect variability on the value of the individual´s time [24]. The differences in overall labor costs, health policy, and other health system factors make challenging to compare results between countries. IBDs as chronic diseases are usually diagnosed in early adulthood causing a severe impact on productivity costs. Even though biologics increase the drug costs, they are assumed to improve the health status and to reduce the burden on resources outside the health care system such as absenteeism from work [67]. Consequently, it is appropriate to include indirect costs in CUA, but indirect costs should be presented separately from direct costs [24,26].

When evaluating effectiveness, it is scientifically and ethically important to use the most appropriate alternative treatment as the control group. The comparator with a good efficacy and safety profile should act as the most cost-effective alternative treatment and is usually the intervention most used in clinical practice [32,68]. However, recommendations on the appropriate comparator vary across countries and depend on the research question [68]. A majority of the included studies used the “standard care” or “usual care” as the comparator.

Source of effectiveness data has substantial influence on model results. RCT data was used as effectiveness data in all included CUAs. RCTs give information about efficacy determined in ideal circumstances and cause a risk of overestimating effectiveness in comparison with the treatment in routine healthcare. Therefore, it is plausible to assume that the CUAs using RCTs as a source of effectiveness produce lower ICERs than real-world data. Contrary to that assumption, only one of the included studies derived information from real life studies and resulted in more favorable ICERs when using response rates from real life data rather than from RCTs [54]. However, the uncertainty in economic evaluations, especially in modeling studies, can arise from numerous methodological disagreements among analyses. Uncertainty caused by e.g., using multiple data sources and extrapolation beyond the time horizon of the study involving the use of assumptions was tested by sensitivity analysis in most studies.

In most studies, the source of utility data was reported inadequately and considerable variation existed in the instruments used to collect it. Direct elicitation methods (e.g., SG, TTO) were used more frequently than indirect methods (e.g., EQ-5D). With direct methods patients directly score their preferences for health states and make judgments based on their own relative values, while indirect methods are based on the patients’ responses to surveys about various aspects of health states [25]. The methods of direct elicitation can be complex and time consuming. In most cases, indirect utility estimates were obtained by determining the relationship between values on a disease-specific measure to a generic quality of life measure. This is necessary because of the fact that the generic measures have been applied in few studies, while disease specific measures such as CDAI are commonly used in RCTs. The application of different algorithms for conversions creates a further source of heterogeneity in ICER estimates. Because of the variation in the methods used and in the preferences across individuals, the QALYs may vary widely between the studies and this affects the results of the CUAs.

Based on previous literature, studies with longer time horizon produce more favorable ICERs than studies with shorter time horizons [49,53]. As biologics improve patients´ health status [67], they have potential to yield gain in terms of reductions in hospitalization, surgeries, and incapacity in future. However, the correlation between the length of the time horizon and cost-effectiveness analyses remains unclear in our study. Although the lifetime horizon is appropriate to capture all health effects and costs for chronic diseases, in most modelling studies the time horizon was limited to one year by the availability of the relevant data and to avoid the bias caused by extrapolation to a longer time horizon.

When considering the previously published systematic reviews, only one study used the standardized quality assessment checklist to evaluate the quality of the included CUAs. As far as we know, this is the first systematic review assessing the quality of economic evaluations by three different checklists. Drummond’s checklist is recommended to inform appraisal of the methodological quality of full economic evaluations [32,69]. Drummond’s checklist is relevant but not sufficient for modeling studies. Therefore, the modeling’s were also assessed using Philip’s checklist [32,34,69]. The CHEERS guideline includes additional items relating to the author’s disclosure of funding sources and conflicts of interest, sufficient information in article titles, and structured abstracts [33]. The CHEERS guideline evaluates the reporting of the study while Drummond’s checklist and Philips’ checklist are designed to assess the methodological quality of economic evaluations.

The amount of the fulfilled items according to Drummond’s checklist and the CHEERS guideline was higher than using Philips’ checklist. The reasons may be aims of the checklist and the extensiveness of Philips’ checklist including several topics relevant to modeling studies and not considered in Drummond’s checklist and the CHEERS guideline. On average, the same CUAs fulfilled the highest amount of the applicable items according to all three checklists. Most of the studies, which fulfilled most criteria of quality assessment checklists, were HTA reports. Almost half of the included CUAs were funded by the pharmaceutical company or authors had received funding from the pharmaceutical companies during the research project [35,36,42,45,47–49,53,55,56]. Many of the studies funded by the pharmaceutical company produced favorable ICERs [35,36,48,49,53,56]. However, it remained unclear whether the source of funding had an effect on the study results. In addition, the relation between the studies funded by a pharmaceutical company and fulfillment of applicable quality assessment criteria was found to be unclear.

The current review was carefully designed beforehand and documented transparently, improving the validity of the study. The study selection, the data extraction, and the quality assessment were performed by one assessor and any ambiguity was resolved with a second assessor to avoid human mistakes and to improve the reliability of the study. The comprehensive literature search was utilized to minimize bias. The intervention treatments included in the search strategy were limited to biologics that had been granted a marketing authorization by the EMA or FDA for the treatment of IBD. Vedolizumab was not included in the search strategy because its marketing authorization was not granted until the planning and realization of the search strategy was completed.

However, because of a limited amount of available CEAs and some inconsistent results, conclusions remain partially uncertain. Furthermore, variability in data input and heterogeneity in study designs made it challenging to compare studies reliably. To improve the reporting of an individual CEA, it is appropriate to use quality assessment checklists. When using checklists, economic evaluations become more consistent, transparent, and informative. The most important predictors of good cost-effectiveness of the biologics were disease activity, the duration of the biological treatment, and the treatment strategy. Further research is needed to confirm cost-effectiveness in moderate IBD. Future studies should evaluate the cost-effectiveness of all available biologic treatments for IBDs. In addition, CEAs between two different biologics are required to find the most cost-effective treatment strategy for IBD patients.

In conclusion, biologics seem to be cost-effective for the induction treatment of active and severe IBD, but not for the maintenance treatment. Whether there are differences in the cost-effectiveness between biologics remains unclear.

## Supporting Information

S1 File Search strategy. (DOC)

S2 File Studies excluded after full-text assessment. (DOC)

S1 Table Inclusion and exclusion criteria. (DOC)

S2 Table Fulfillment of items of quality assessment checklists. (DOC)

S3 Table PRISMA checklist. (DOC)

## References

[1] Dignass A, Eliakim R, Magro F, Maaser C, Chowers Y, Geboes K, et al Second European evidence-based consensus on the diagnosis and management of ulcerative colitis part 1: definitions and diagnosis. J Crohns Colitis. 2012;6: 965–990. 10.1016/j.crohns.2012.09.003 . 23040452

[2] Van Assche G, Dignass A, Panes J, Beaugerie L, Karagiannis J, Allez M, et al The second European evidence-based Consensus on the diagnosis and management of Crohn’s disease: Definitions and diagnosis. J Crohns Colitis. 2010;4: 7–27. 10.1016/j.crohns.2009.12.003 . 21122488

[3] Molodecky NA, Soon IS, Rabi DM, Ghali WA, Ferris M, Chernoff G, et al Increasing incidence and prevalence of the inflammatory bowel diseases with time, based on systematic review. Gastroenterology. 2012;142: 46–54. 10.1053/j.gastro.2011.10.001 . 22001864

[4] Burisch J, Jess T, Martinato M, Lakatos PL. The burden of inflammatory bowel disease in Europe. J Crohns Colitis. 2013;7: 322–337. 10.1016/j.crohns.2013.01.010 . 23395397

[5] Dignass A, Van Assche G, Lindsay JO, Lémann M, Söderholm J, Colombel JF, et al The second European evidence-based Consensus on the diagnosis and management of Crohn’s disease: Current management. J Crohns Colitis. 2010;4: 28–62. 10.1016/j.crohns.2009.12.002 . 21122489

[6] Dignass A, Lindsay JO, Sturm A, Windsor A, Colombel J-F, Allez M, et al Second European evidence-based consensus on the diagnosis and management of ulcerative colitis part 2: current management. J Crohns Colitis. 2012;6: 991–1030. 10.1016/j.crohns.2012.09.002 . 23040451

[7] Øresland T, Bemelman WA, Sampietro GM, Spinelli A, Windsor A, Ferrante M, et al European evidence-based consensus on surgery for ulcerative colitis. J Crohns Colitis. 2015;9: 4–25. 10.1016/j.crohns.2014.08.012 . 25304060

[8] Ford AC, Sandborn WJ, Khan KJ, Hanauer SB, Talley NJ, Moayyedi P. Efficacy of biological therapies in inflammatory bowel disease: systematic review and meta-analysis. Am J Gastroenterol. 2011;106: 644–659. 10.1038/ajg.2011.73 . 21407183

[9] European Medicines Agency; 2015. Database: European public assessment reports [Internet]. Accessed: http://www.ema.europa.eu/ema/index.jsp?curl=pages/medicines/landing/epar_search.jsp&mid=WC0b01ac058001d124.

[10] U.S. Food and Drug Administration; 2015. Database: Drugs@FDA. FDA Approved Drug Products [Internet]. Accessed: http://www.accessdata.fda.gov/scripts/cder/drugsatfda/.

[11] Odes S, Vardi H, Friger M, Wolters F, Russel MG, Riis L, et al Cost analysis and cost determinants in a European inflammatory bowel disease inception cohort with 10 years of follow-up evaluation. Gastroenterology. 2006;131: 719–728. 10.1053/j.gastro.2006.05.052 . 16952541

[12] Van der Valk ME, Mangen M-JJ, Leenders M, Dijkstra G, van Bodegraven AA, Fidder HH, et al Healthcare costs of inflammatory bowel disease have shifted from hospitalisation and surgery towards anti-TNFα therapy: results from the COIN study. Gut. 2014;63: 72–79. 10.1136/gutjnl-2012-303376 . 23135759

[13] Frolkis AD, Dykeman J, Negrón ME, Debruyn J, Jette N, Fiest KM, et al Risk of surgery for inflammatory bowel diseases has decreased over time: a systematic review and meta-analysis of population-based studies. Gastroenterology. 2013;145: 996–1006. 10.1053/j.gastro.2013.07.041 . 23896172

[14] Lichtenstein GR, Hanauer SB, Sandborn WJ. Management of Crohn’s disease in adults. Am J Gastroenterol. 2009;104: 465–483. 10.1038/ajg.2008.168 . 19174807

[15] Lv R, Qiao W, Wu Z, Wang Y, Dai S, Liu Q, et al Tumor necrosis factor alpha blocking agents as treatment for ulcerative colitis intolerant or refractory to conventional medical therapy: a meta-analysis. PLoS One. 2014;9: e86692 10.1371/journal.pone.0086692 . 24475168 PMC3903567

[16] Lawson M, Thomas A, Akobeng A. Tumour necrosis factor alpha blocking agents for induction of remission in ulcerative colitis. Cochrane Database Syst Rev. 2006; 1–29. 10.1002/14651858.CD005112.pub2 16856078

[17] Danese S, Vuitton L, Peyrin-Biroulet L. Biologic agents for IBD: practical insights. Nat Rev Gastroenterol Hepatol 2015 12: 537–545. 10.1038/nrgastro.2015.135 . 26284562

[18] Tang DH, Harrington AR, Lee JK, Lin M, Armstrong EP. A systematic review of economic studies on biological agents used to treat Crohn’s disease. Inflamm Bowel Dis. 2013;19: 2673–2694. 10.1097/MIB.0b013e3182916046 . 23792552

[19] Marshall J, Blackhouse G, Goeree R, Brazier N, Irvine E, Faulkner L, et al. Infliximab for the treatment of Crohn’s disease: A systematic review and cost-utility analysis [Technology report no 24]. Ottawa: Canadian Coordinating Office for Health Technology Assessment (CCOHTA). 2002. Available: https://www.cadth.ca/sites/default/files/pdf/122_infliximab_tr_e.pdf. Accessed 1 July 2015.

[20] Fleurence R, Spackman E. Cost-effectiveness of biologic agents for treatment of autoimmune disorders: structured review of the literature. J Rheumatol. 2006;33: 2124–2131. 17086602

[21] Dretzke J, Edlin R, Round J, Connock M, Hulme C, Czeczot J, et al A systematic review and economic evaluation of the use of tumour. Health Technol Assess. 2011;15: 1–252. 10.3310/hta15060 . 21291629 PMC4781107

[22] Assasi N, Blackhouse G, Xie F, Gaebel K, Marshall J, Irvine EJ, et al. Anti-TNF-alfa drugs for refractory inflammatory bowel disease: Clinical- and cost-effectiveness analyses [Technology Report no 120]. Ottawa: Canadian Agency for Drugs and Technologies in Health (CADTH). 2009. Available: https://www.cadth.ca/media/pdf/H0479_Anti_TNF_a_Drugs_for_Refractory_Inflammatory_Bowel_Disease_tr_e.pdf. Accessed 1 July 2015.

[23] Torrance G, Siegel J, Luce B. Framing and Designing the Cost-Effectiveness Analysis In: Gold MR, Siegel JE, Russell LB, Weinstein MC, editors. Cost-Effectiveness in Health and Medicine. New York: Oxford University of Press; 1996 pp. 54–81.

[24] Drummond MF, Sculpher MJ, Torrance GW, O´Brien BJ, Stoddart GL. Methods for the Economic Evaluation of Health Care Programmes. 3rd ed New York: Oxford University Press; 2005.

[25] Gold M, Patrick D, Torrance G, Fryback D, Hadorn D, Kamlet M, et al Identifying and Valuing Outcomes In: Gold MR, Siegel JE, Russell LB, Weinstein MC, editors. Cost-Effectiveness in Health and Medicine. New York: Oxford University of Press; 1996 pp. 82–134.

[26] Luce B, Manning W, Siegel J, Lipscomb J. Estimating Costs in Cost-Effectiveness Analysis In: Gold MR, Siegel JE, Russell LB, Weinstein MC, editors. Cost-Effectiveness in Health and Medicine. New York: Oxford University of Press; 1996 pp. 176–213.

[27] Methods Guide for Medical Test Reviews. AHRQ Publication no 12-EC017. Rockville: Agency for Healthcare Research and Quality; 2012. Available: http://www.effectivehealthcare.ahrq.gov/ehc/products/246/558/Methods-Guide-for-Medical-Test-Reviews_Full-Guide_20120530.pdf. Accessed 1 July 2015.

[28] Shemilt I, Mugford M, Byford S, Drummond M, Eisenstein E, Knapp M, et al Chapter 15: Incorporating economics evidence In: Higgins JPT, Green S, editors. Cochrane Handbook for Systematic Reviews of Interventions; 2008 pp. 447–479.

[29] Craig D, Rice S. NHS Economic Evaluation Database Handbook. 3rd ed York: Centre for Reviews and Dissemination, University of York; 2007.

[30] European Central Bank. Bilateral Exchange rates; 2015. Database: Statistical Data Warehouse [Internet]. Accessed: https://sdw.ecb.europa.eu/browse.do?node=2018794.

[31] Official Statistics of Finland. Value of Money 1860–2014. Database: Consumer price index 2014 [Internet]. Helsinki, Finland: Satatistics of Finalnd; 2014. Acessed: http://www.stat.fi/til/khi/2014/khi_2014_2015-01-19_tau_001.html.

[32] Drummond M, Jefferson T. Guidelines for authors and peer reviewers of economic submissions to the BMJ. BMJ. 1996;313: 275–283. 10.1136/bmj.313.7052.275 . 8704542 PMC2351717

[33] Husereau D, Drummond M, Petrou S, Carswell C, Moher D, Greenberg D, et al Consolidated Health Economic Evaluation Reporting Standards (CHEERS)—explanation and elaboration: a report of the ISPOR Health Economic Evaluation Publication Guidelines Good Reporting Practices Task Force. Value Health. 2013;16: 231–250. 10.1016/j.jval.2013.02.002 . 23538175

[34] Philips Z, Ginnelly L, Sculpher M, Claxton K. Review of guidelines for good practice in decision-analytic modelling in health technology assessment. Health Technol Assess. 2004;8: 1–172. 10.3310/hta8360 15361314

[35] Punekar YS, Hawkins N. Cost-effectiveness of infliximab for the treatment of acute exacerbations of ulcerative colitis. Eur J Health Econ. 2010;11: 67–76. 10.1007/s10198-009-0199-5 . 19844750

[36] Chaudhary MA, Fan T. Cost-Effectiveness of Infliximab for the Treatment of Acute Exacerbations of Ulcerative Colitis in the Netherlands. Biol Ther. 2013;3: 45–60. 10.1007/s13554-012-0007-0 . 24392304 PMC3873082

[37] Bryan S, Andronis L, Hyde C, Connock M, Fry-Smith A, Wang D. Infliximab for the treatment of acute exacerbations of ulcerative colitis. Evidence Review Group Report commissioned by the NHS R&D HTA Programme on behalf of NICE. 2008:1–120. Available: http://www.nets.nihr.ac.uk/__data/assets/pdf_file/0019/82513/ERGReport-08-37-01.pdf. Accessed 1 July 2015.

[38] Hyde C, Bryan S, Biddle K, Massey A. Infliximab for ulcerative colitis. Evidence Review Group Report commissioned by the NHS R&D HTA Programme on behalf of NICE. 2007:1–113. Available: http://www.nets.nihr.ac.uk/__data/assets/pdf_file/0003/82506/ERGReport-07-12-01.pdf. Accessed 1 July 2015.

[39] Ananthakrishnan AN, Hur C, Juillerat P, Korzenik JR. Strategies for the prevention of postoperative recurrence in Crohn’s disease: results of a decision analysis. Am J Gastroenterol. 2011;106: 2009–2017. 10.1038/ajg.2011.237 . 21788991

[40] Ananthakrishnan AN, Hur C, Korzenik JR. Certolizumab pegol compared to natalizumab in patients with moderate to severe Crohn’s disease: results of a decision analysis. Dig Dis Sci. 2012;57: 472–480. 10.1007/s10620-011-1896-3 . 21909990

[41] Arseneau KO, Cohn SM, Cominelli F, Connors AF. Cost-utility of initial medical management for Crohn’s disease perianal fistulae. Gastroenterology. 2001;120: 1640–1656. 11375946 10.1053/gast.2001.24884

[42] Blackhouse G, Assasi N, Xie F, Marshall J, Irvine EJ, Gaebel K, et al Canadian cost-utility analysis of initiation and maintenance treatment with anti-TNF-α drugs for refractory Crohn’s disease. J Crohns Colitis. 2012;6: 77–85. 10.1016/j.crohns.2011.07.007 . 22261531

[43] Bodger K, Kikuchi T, Hughes D. Cost-effectiveness of biological therapy for Crohn’s disease: Markov cohort analyses incorporating United Kingdom patient-level cost data. Aliment Pharmacol Ther. 2009;30: 265–274. 10.1111/j.1365-2036.2009.04033.x . 19438428

[44] Clark W, Raftery J, Song F, Barton P, Cummins C, Fry-Smith A, et al Systematic review and economic evaluation of the effectiveness of infliximab for the treatment of Crohn’s disease. Health Technol Assess. 2003;7: 1–80. . 12709295 10.3310/hta7030

[45] Doherty G, Miksad R, Cheifetz A, Moss A. Comparative cost-effectiveness of strategies to prevent postoperative clinical recurrence of Crohn’s disease. Inflamm Bowel Dis. 2012;18: 1608–1616. 10.1002/ibd.21904 21905173 PMC3381977

[46] Jaisson-hot I, Flourie B, Descos L, Colin C. Management for severe Crohn’s disease : A lifetime cost-utility analysis. Int J Technol Assess Health Care. 2004;20: 274–279. 15446756 10.1017/s0266462304001084

[47] Kaplan GG, Hur C, Korzenik J, Sands BE. Infliximab dose escalation vs. initiation of adalimumab for loss of response in Crohn’s disease: a cost-effectiveness analysis. Aliment Pharmacol Ther. 2007;26: 1509–1520. 10.1111/j.1365-2036.2007.03548.x . 17931345

[48] Lindsay J, Punekar YS, Morris J, Chung-Faye G. Health-economic analysis: cost-effectiveness of scheduled maintenance treatment with infliximab for Crohn’s disease-modelling outcomes in active luminal and fistulizing disease in adults. Aliment Pharmacol Ther. 2008;28: 76–87. 18410558 10.1111/j.1365-2036.2008.03709.x

[49] Loftus E V, Johnson SJ, Yu AP, Wu EQ, Chao J, Mulani PM. Cost-effectiveness of adalimumab for the maintenance of remission in patients with Crohn’s disease. Eur J Gastroenterol Hepatol. 2009;21: 1302–1309. 10.1097/MEG.0b013e32832a8d71 . 19465858

[50] Marchetti M, Liberato NL, Di Sabatino A, Corazza GR. Cost-effectiveness analysis of top-down versus step-up strategies in patients with newly diagnosed active luminal Crohn’s disease. Eur J Heal Econ. 2013;14: 853–861. 10.1007/s10198-012-0430-7 . 22975794

[51] Saito S, Shimizu U, Nan Z, Mandai N, Yokoyama J, Terajima K, et al Economic impact of combination therapy with infliximab plus azathioprine for drug-refractory Crohn’s disease: a cost-effectiveness analysis. J Crohns Colitis. 2013;7: 167–174. 22626508 10.1016/j.crohns.2012.04.007

[52] Tang DH, Armstrong EP, Pharm D, Lee JK. Cost-Utility Analysis of Biologic Treatments for Moderate-to-Severe Crohn’s Disease. Pharmacotherapy. 2012;32: 515–526. 10.1002/j.1875-9114.2011.01053.x . 22528603

[53] Tsai HH, Punekar YS, Morris J, Fortun P. A model of the long-term cost effectiveness of scheduled maintenance treatment with infliximab for moderate-to-severe ulcerative colitis. Aliment Pharmacol Ther. 2008;28: 1230–1239. 18729845 10.1111/j.1365-2036.2008.03839.x

[54] Ung V, Thanh NX, Wong K, Kroeker KI, Lee T, Wang H, et al Real-life Treatment Paradigms Show Infliximab Is Cost-effective for Management of Ulcerative Colitis. Clin Gastroenterol Hepatol. 2014;12: 1871–1878. 10.1016/j.cgh.2014.03.012 . 24674943

[55] Xie F, Blackhouse G, Assasi N, Gaebel K, Robertson D, Goeree R. Cost-utility analysis of infliximab and adalimumab for refractory ulcerative colitis. Cost Eff Resour Alloc. 2009;7: 1–8. 10.1186/1478-7547-7-20 . 20003364 PMC2797497

[56] Yu AP, Johnson S, Wang S, Atanasov P, Tang J, Wu E, et al Cost Utility of Adalimumab versus Infliximab Maintenance Therapies in the United States for Moderately to Severely Active Crohn’s Disease. 2009;27: 609–621. 10.2165/11312710-000000000-00000 . 19663531

[57] Casellas F, Arenas JI, Baudet JS, Fa S, Gelabert J, Medina C, et al Impairment of Health-related Quality of Life in Patients with Inflammatory Bowel Disease: A Spanish Multicenter Study. Inflamm Bowel Dis. 2005;11: 488–496. . 15867589 10.1097/01.mib.0000159661.55028.56

[58] Gregor J, McDonald J, Klar N, Wall R, Atkinson K, Lamba B, et al An evaluation of utility measurement in Crohn’s disease. Inflamm Bowel Dis. 1997;3: 265–276. 23282873

[59] Buxton M, Lacey L, Feagan B, Niecko T, Miller D, Townsend R. Mapping from disease-specific measures to utility: an analysis of the relationships between the Inflammatory Bowel Disease Questionnaire and Crohn’s Disease Activity Index in Crohn’s disease and measures of utility. Value Heal. 2007;10: 214–220. 10.1111/j.1524-4733.2007.00171.x . 17532814

[60] Paul D. Modeling Valuations for EuroQol Health States. Med Care. 1997;35: 1095–1108. 9366889 10.1097/00005650-199711000-00002

[61] Arseneau KO, Sultan S, Provenzale DT, Onken J, Bickston SJ, Foley E, et al Do patient preferences influence decisions on treatment for patients with steroid-refractory ulcerative colitis? Clin Gastroenterol Hepatol. 2006;4: 1135–1142. 16829206 10.1016/j.cgh.2006.05.003

[62] National Institute for Health and Care Excellence [Internet]. Guide to the methods of technology appraisal 2013. 2013. Available: http://publications.nice.org.uk/guide-to-the-methods-of-technology-appraisal-2013-pmg9. Accessed 1 July 2015. 27905712

[63] Rocchi A, Menon D, Verma S, Miller E. The Role of Economic Evidence in Canadian Oncology Reimbursement Decision-Making: To Lambda and Beyond. Value Heal. 2008;11: 771–783. 10.1111/j.1524-4733.2007.00298.x . 18179658

[64] Shiroiwa T, Sung Y, Fukuda T, Lang H. International Survey on Willingness-to-Pay (WTP) for one Additional QALY Gained: What is the Threshold of Cost Effectiveness? Health Econ. 2010;437: 422–437. 10.1002/hec.1481 19382128

[65] Kaplan RM, Bush JW. Health-Related Quality of Life Measurement for Evaluation Research and Policy Analysis. Heal Psychol. 1982;1: 61–80.

[66] World Health Organisation [Internet]. Cost-Effectiveness thresholds. 2015. Available: http://www.who.int/choice/costs/CER_thresholds/en/. Accessed 1 July 2015.

[67] Lichtenstein GR, Yan S, Bala M, Hanauer S. Remission in Patients with Crohn’s Disease is Associated with Improvement in Employment and Quality of Life and a Decrease in Hospitalizations and Surgeries. Am J Gastroenterol. 2004; 99:91–96. 14687148 10.1046/j.1572-0241.2003.04010.x

[68] European Network for Health Technology Assessment (EUnetHTA) [Internet]. Criteria for the choice choice of the most appropriate comparator(s). Summary of current policies and best practice recommendations. February, 2013. Available: http://www.eunethta.eu/sites/5026.fedimbo.belgium.be/files/Choice_of_comparator.pdf. Accessed 1 July 2015.

[69] Higgins JPT, Altman DG. Chapter 8: Assessing risk of bias in included studies In: Higgins JPT, Green S, editors. Cochrane Handbook for Systematic Reviews of Interventions; 2008 pp. 187–241.

