# Supplementary material for: A Systematic Review of the Cost-Effectiveness of Biologics for the Treatment of Inflammatory Bowel Diseases
Source: PLoS One. 2015 Dec 16;10(12):e0145087. doi: 10.1371/journal.pone.0145087 (PMC4682717; doi:10.1371/journal.pone.0145087)
Supplement: S1 File — (DOC) [file pone.0145087.s001.doc]

**S1 File. Search strategy.**

Search strategy for Medline (Ovid) and Medline In-Process & Non-Indexed Citations (Ovid)

Date of the search June 7, 2014

(inflammatory bowel diseases/ OR crohn disease/ OR colitis/ OR colitis, ulcerative/ OR inflammatory bowel disease*.mp. OR ibd*.mp. OR crohn*.mp. OR regional enteritis.mp. OR granulomatous enteritis.mp. OR ileitis.mp. OR colitis.mp.) AND (tumor necrosis factor-alpha/ OR tumo?r necrosis factor*.mp. OR anti-tumo?r necrosis factor*.mp. OR antitumor?r necrosis factor*.mp. OR tnf*.mp. OR antitnf*.mp. OR antitnf*. mp. OR biologics.mp. OR biologic agent*.mp. OR biologic therap*.mp. OR infliximab.mp. OR remicade*.mp. OR inflectra*.mp. OR remsima*.mp. OR ca2*.mp. OR adalimumab.mp. OR humira*.mp. OR trudexa*.mp. OR d2e7*.mp. OR certolizumab.mp. OR cimzia*.mp. OR cdp870*.mp. OR cdp 870*.mp. OR pha738144*.mp. OR pha 738144*.mp. OR golimumab.mp. OR simponi*.mp. OR cnto148*.mp. OR cnto 148*.mp. OR integrin alpha4/ OR naralizumab.mp. OR tysabri*.mp. OR antegren*.mp. OR (integrin* ADJ alpha4*).mp. OR (integrin* ADJ “alpha 4*”).mp. OR (anti ADJ2 “4 integrin*”).mp. OR cd49d*.mp.) AND (Economics/ OR exp “Costs and Cost Analysis”/ OR exp Economics, Hospital/ OR exp Economics, Medical/ OR Economics, Pharmaceutical/ OR exp “Fees and Charges”/ OR econom*.mp. OR cost*.mp. OR price*.mp. OR pricing*.mp. OR expense*.mp. OR Quality-Adjusted Life Years/ OR health related quality of life*.mp. OR hrqol*.mp. OR quality adjusted life year*.mp. OR qaly*.mp. OR utility.mp. OR eq5d*.mp. OR eq 5d*.mp. OR euroqol*.mp. OR euro qol*.mp. OR euroqual*.mp. OR euro qual*.mp. OR 15d*.mp. OR sf36*.mp. OR sf6d*.mp. OR (shortform AND (85 OR thirtysix OR “thirty six” OR 6d OR six*)).mp. OR (“short form” AND (85 OR thirtysix OR “thirty six” OR 6d OR six*)).mp. OR (sf AND (85 OR thirtysix OR “thirty six” OR 6d OR six*)).mp. OR aqol*.mp. OR assessment of quality of life*.mp. OR hui*.mp. OR health utilit* index.mp. OR qwb*.mp. OR quality of well-being*.mp. OR vas.mp. OR visual analog.mp. OR time trade-off.mp. OR standard gamble.mp.)
