# Supplementary material for: A Systematic Review of the Cost-Effectiveness of Biologics for the Treatment of Inflammatory Bowel Diseases
Source: PLoS One. 2015 Dec 16;10(12):e0145087. doi: 10.1371/journal.pone.0145087 (PMC4682717; doi:10.1371/journal.pone.0145087)
Supplement: S2 File — (DOC) [file pone.0145087.s002.doc]

**S2 File. Studies excluded after full-text assessment.**

*Inclusion criteria not met (n =28)*

Afif W, Loftus EV,Jr, Faubion WA, Kane SV, Bruining DH, Hanson KA, et al. Clinical utility of measuring infliximab and human anti-chimeric antibody concentrations in patients with inflammatory bowel disease. Am J Gastroenterol. 2010;105: 1133-1139.

Ananthakrishnan AN, Korzenik JR, Hur C. Can mucosal healing be a cost-effective endpoint for biologic therapy in Crohn's disease? A decision analysis. Inflamm Bowel Dis. 2013;19: 37-44.

Bakhshai J, Bleu-Laine R, Jung M, Lim J, Reyes C, Sun L, et al. The cost effectiveness and budget impact of natalizumab for formulary inclusion. J Med Econ. 2010;13: 63-69.

Bell S, Kamm MA. Anti-TNF-alpha antibody treatment for Crohn's disease. Research and Clinical Forums. 2000;22: 129-135.

Bernstein CN. Infliximab as first-line therapy for Crohn's disease. Inflamm Bowel Dis. 2002;8: 63-65.

Bodger K. Cost effectiveness of treatments for inflammatory bowel disease. Pharmacoeconomics. 2011;29: 387-401.

Bodger K. Economic implications of biological therapies for Crohn's disease: review of infliximab. Pharmacoeconomics. 2005;23: 875-888.

Carter CT, Waters HC, Smith DB. Impact of infliximab adherence on Crohn's disease-related healthcare utilization and inpatient costs. Adv Ther. 2011;28: 671-683.

Choi GK, Collins SD, Greer DP, Warren L, Dowson G, Clark T, et al. Costs of adalimumab versus infliximab as first-line biological therapy for luminal Crohn's disease. J Crohns Colitis. 2014;8: 375-383.

Cohen RD, Thomas T. Economics of the use of biologics in the treatment of inflammatory bowel disease. Gastroenterol Clin North Am. 2006;35: 867-882.

Di Sabatino A, Liberato L, Marchetti M, Biancheri P, Corazza GR. Optimal use and cost-effectiveness of biologic therapies in inflammatory bowel disease. Intern emerg medicine. 2011;6: 17-27.

Dubinsky MC, Reyes E, Ofman J, Chiou C-, Wade S, Sandborn WJ. A cost-effectiveness analysis of alternative disease management strategies in patients with Crohn's disease treated with azathioprine or 6-mercaptopurine. Am J Gastroenterol. 2005;100: 2239-2247.

Fleurence R, Spackman E. Cost-effectiveness of biologic agents for treatment of autoimmune disorders: structured review of the literature. J Rheumatol. 2006;33: 2124–2131.

Karmiris K, Koutroubakis IE. Role of infliximab in the treatment of fistulizing Crohn's disease. Annals of Gastroenterology. 2005;18: 297-302.

Koelewijn C, Schrijvers A, Oldenburg B. Infliximab use in patients with Crohn's disease: quality of life, costs and resource use. Neth J Med. 2006;64: 212-218.

Lee JK, Tang DH, Mollon L, Armstrong EP. Cost-effectiveness of biological agents used in ulcerative colitis. Baillieres Best Pract Res Clin Gastroenterol. 2013;27: 949-960.

Lindsay JO, Chipperfield R, Giles A, Wheeler C, Orchard T, INDIGO study i. A UK retrospective observational study of clinical outcomes and healthcare resource utilisation of infliximab treatment in Crohn's disease. Aliment Pharmacol Ther. 2013;38: 52-61.

Liu Y, Wu EQ, Bensimon AG, Fan CP, Bao Y, Ganguli A, et al. Cost per responder associated with biologic therapies for Crohn's disease, psoriasis, and rheumatoid arthritis. Adv Ther. 2012;29: 620-634.

Louis E, Lofberg R, Reinisch W, Camez A, Yang M, Pollack PF, et al. Adalimumab improves patient-reported outcomes and reduces indirect costs in patients with moderate to severe Crohn's disease: results from the CARE trial. J Crohns Colitis. 2013;7: 34-43.

Nahar IK, Shojania K, Marra CA, Alamgir AH, Anis AH. Infliximab treatment of rheumatoid arthritis and Crohn's disease. Ann Pharmacother. 2003;37: 1256-1265.

Norum J, Koldingsnes W, Aanes T, Antonsen MA, Florholmen J, Kondo M. The economic burden of TNFalpha inhibitors and other biologic treatments in Norway. ClinicoEcon outcomes res. 2011;3: 73-78.

Odes S. How expensive is inflammatory bowel disease? A critical analysis. World J Gastroenterol. 2008;14: 6641-6647.

Park KT, Bass D. Inflammatory bowel disease-attributable costs and cost-effective strategies in the United States: a review. Inflamm Bowel Dis. 2011;17: 1603-1609.

Park KT, Crandall WV, Fridge J, Leibowitz IH, Tsou M, Dykes DM, et al. Implementable strategies and exploratory considerations to reduce costs associated with anti-TNF therapy in inflammatory bowel disease. Inflamm Bowel Dis. 2014;20: 946-951.

Park KT, Tsai R, Perez F, Cipriano LE, Bass D, Garber AM. Cost-effectiveness of early colectomy with ileal pouch-anal anastamosis versus standard medical therapy in severe ulcerative colitis. Ann Surg. 2012;256: 117-124.

Tang DH, Harrington AR, Lee JK, Lin M, Armstrong EP. A systematic review of economic studies on biological agents used to treat Crohn’s disease. Inflamm Bowel Dis. 2013;19: 2673–2694.

Yen EF, Kane SV, Ladabaum U. Cost-effectiveness of 5-aminosalicylic acid therapy for maintenance of remission in ulcerative colitis. Am J Gastroenterol. 2008;103: 3094-3105.

Zisman TL, Cohen RD. Pharmacoeconomics and quality of life of current and emerging biologic therapies for inflammatory bowel disease. Curr Treat Options Gastroenterol. 2007;10: 185-194.

*No English full-text available (n =3)*

Rudakova AV. [Cost-effectiveness of tumor necrosis factor in Crohn's disease]. Eksp Klin Gastroenterol. 2012: 83-86.

Rudakova AV. [The cost effectiveness of adalimumab in Crohn's disease (review of foreign pharmacoeconomic studies)]. Eksp Klin Gastroenterol. 2011: 113-119.

Zhu WM, Zuo LG, Li Y, Cao L, Zhang W, Gu YF, et al. [A comparative study of induction of remission and cost-effectiveness of enteral nutrition versus infliximab in moderate-to-severe Crohn's disease]. Chung Hua Nei Ko Tsa Chih. 2013;52: 721-725.
