# Supplementary material for: A Systematic Review of the Cost-Effectiveness of Biologics for the Treatment of Inflammatory Bowel Diseases
Source: PLoS One. 2015 Dec 16;10(12):e0145087. doi: 10.1371/journal.pone.0145087 (PMC4682717; doi:10.1371/journal.pone.0145087)
Supplement: S1 Table — (DOC) [file pone.0145087.s003.doc]

**S1 Table. Inclusion and exclusion criteria.**

|  | **Inclusion criteria** | **Exclusion criteria** |
| --- | --- | --- |
| Patient | ≥ 16-year old patients  Diagnosed moderate-to-severe CD or UC | < 16-year old patients  Diagnosed mild CD or UC  No diagnosed CD or UC |
| Intervention | Biological treatment alone or together with conventional treatment or surgery | No biologic treatment |
| Comparison | Conventional treatment, surgery, biological treatment or placebo treatment | No comparative treatment |
| Outcome | ICER^a^ | Only costs or effectiveness |
| Time horizon | ≥ 12 months | < 12 months |
| Study design | Cost-effectiveness analyze  Cost-effectiveness analyze by modelling | Cost-minimization analyze  Cost-benefit analyze  No health economic evaluation  Published only as an abstract  No English full-text |
| CD, Crohn´s Disease; QALY, Quality-Adjusted Life Year; ICER, Incremental cost-effectiveness ratio; UC, Ulcerative Colitis.  ^a^The difference in costs divided by the difference in health effects. | | |
