# Supplementary material for: A Systematic Review of the Cost-Effectiveness of Biologics for the Treatment of Inflammatory Bowel Diseases
Source: PLoS One. 2015 Dec 16;10(12):e0145087. doi: 10.1371/journal.pone.0145087 (PMC4682717; doi:10.1371/journal.pone.0145087)
Supplement: S2 Table — (DOC) [file pone.0145087.s004.doc]

**S2 Table. Fulfillment of items of quality assessment checklists.**

|  | Ananthakrishnan et al 2011 | Ananthakrishnan et al 2012 | Arseneau et al 2001 | Assasi et al 2009 | Blackhouse et al 2012 | Bodger et al 2009 | Bryan et al 2008 | Clark et al 2003 | | Dohertyet al 2012 | | Dretzke et al 2011 | | Jaisson-Hot et al 2004 | | Kaplan et al 2007 | | Lindsay et al 2008 | | Loftus et al 2009 | | Marchetti et al 2013 | | Marshall et al 2002 | | Mohammad et al 2013 | | Punekar et al 2010 | | Saito et al 2013 | | Tanget al 2012 | | Tsai et al 2008 | | Ung et al 2014 | | Xie et al 2009 | | Yu et al 2009 |
| --- | --- | --- | --- | --- | --- | --- | --- | --- | --- | --- | --- | --- | --- | --- | --- | --- | --- | --- | --- | --- | --- | --- | --- | --- | --- | --- | --- | --- | --- | --- | --- | --- | --- | --- | --- | --- | --- | --- | --- | --- |
| **Drummond´s checklist** | | | | | | | | | | | | | | | | | | | | | | | | | | | | | | | | | | | | | | | | |
| Study design | | | | | | | | | | | | | | | | | | | | | | | | | | | | | | | | | | | | | | | | |
| 1.The research question is stated | + | + | + | + | + | + | + | + | | + | | + | | + | | + | | + | | + | | + | | + | | + | | + | | + | | + | | + | | + | | + | | + |
| 2. The economic importance of the research question is stated. | + | + | + | + | + | + | + | + | | + | | + | | + | | + | | + | | + | | + | | + | | - | | + | | + | | + | | + | | + | | + | | + |
| 3. The viewpoint(s) of the analysis are clearly stated and justified. | + | + | + | + | + | + | + | - | | + | | + | | + | | - | | + | | + | | + | | + | | + | | + | | + | | + | | + | | + | | + | | + |
| 4. The rationale for choosing alternative programmes or interventions compared is stated. | + | + | + | + | + | + | + | + | | - | | + | | + | | + | | + | | + | | + | | + | | + | | + | | - | | + | | + | | + | | + | | + |
| 5. The alternatives being compared are clearly described. | + | + | + | - | - | + | + | - | | + | | + | | + | | + | | - | | - | | + | | + | | + | | + | | + | | + | | - | | + | | - | | + |
| 6. The form of economic evaluation used is stated. | + | + | + | + | + | + | + | + | | + | | + | | + | | + | | + | | + | | + | | + | | + | | + | | + | | + | | + | | + | | + | | + |
| 7. The choice of form of economic evaluation is justified in relation to the questions addressed. | + | + | + | + | + | + | + | a)+ b)-  c)- | | + | | + | | + | | + | | + | | - | | + | | + | | + | | + | | + | | + | | + | | + | | + | | - |
| Data exctraction | | | | | | | | | | | | | | | | | | | | | | | | | | | | | | | | | | | | | | | | |
| 8. The source(s) of effectiveness estimates used are stated. | + | + | + | + | + | + | + | + | | + | | + | | + | | + | | + | | + | | + | | + | | + | | + | | + | | + | | + | | + | | + | | + |
| 9. Details of the design and results of effectiveness study are given (if based on a single study). | Na | Na | Na | Na | Na | Na | Na | a) Na  b)Na  c)- | | Na | | Na | | + | | Na | | Na | | Na | | + | | Na | | Na | | Na | | Na | | Na | | Na | | Na | | Na | | Na |
| 10. Details of the methods of synthesis or meta-analysis of estimates are given (if based on a synthesis of a number of effectiveness studies). | - | - | + | + | + | + | + | a)-  b)-  c)Na | | + | | + | | Na | | - | | - | | - | | Na | | + | | - | | + | | - | | - | | - | | - | | + | | - |
| 11. The primary outcome measure(s) for the economic evaluation are clearly stated. | + | + | + | + | + | + | + | + | | + | | + | | + | | + | | + | | + | | + | | + | | + | | + | | + | | + | | + | | + | | + | | + |
| 12. Methods to value benefits are stated. | + | + | + | + | + | + | + | + | | + | | + | | + | | + | | + | | + | | + | | + | | + | | + | | + | | + | | + | | + | | + | | + |
| 13. Details of the subjects from whom valuations were obtained were given. | + | + | + | + | + | + | + | + | | + | | + | | + | | + | | + | | + | | + | | + | | + | | + | | + | | + | | + | | + | | + | | + |
| 14. Productivity changes (if included) are reported separately. | Na | Na | Na | Na | Na | Na | Na | Na | | - | | Na | | Na | | Na | | Na | | + | | Na | | Na | | Na | | Na | | Na | | Na | | Na | | Na | | Na | | Na |
| 15. The relevance of productivity changes to the study question is discussed. | - | - | + | + | + | - | - | - | | - | | - | | + | | - | | + | | + | | + | | + | | + | | - | | - | | - | | + | | - | | - | | + |
| 16. Quantities of resource use are reported separately from their unit costs. | - | - | + | + | + | - | + | - | | - | | + | | - | | - | | - | | - | | - | | + | | + | | + | | - | | + | | + | | - | | + | | + |
| 17. Methods for the estimation of quantities and unit costs are described. | + | + | + | + | + | + | + | - | | - | | + | | + | | + | | + | | + | | + | | + | | + | | + | | + | | + | | + | | + | | + | | + |
| 18. Currency and price data are recorded. | + | + | + | + | + | - | + | - | | + | | + | | + | | + | | + | | + | | + | | + | | + | | + | | + | | + | | + | | - | | + | | + |
| 19. Details of currency of price adjustments for inflation or currency conversion are given. | + | + | + | CD) –  UC)+ | - | + | + | - | | + | | + | | - | | + | | - | | + | | - | | + | | + | | + | | + | | + | | - | | + | | + | | + |
| 20. Details of any model used are given. | + | + | + | + | + | + | + | - | | + | | + | | + | | + | | + | | - | | + | | + | | + | | + | | + | | + | | + | | + | | + | | - |
| 21. The choice of model used and the key parameters on which it is based are justified. | + | + | + | + | + | + | + | a)+  b)-  c)- | | + | | + | | + | | + | | + | | - | | + | | + | | + | | + | | + | | + | | + | | + | | + | | - |
| Analysis and interpretation of results | | | | | | | | | | | | | | | | | | | | | | | | | | | | | | | | | | | | | | | | |
| 22. Time horizon of costs and benefits is stated. | + | + | + | + | + | + | + | a)+  b)-  c)+ | | + | | + | | + | | + | | + | | + | | + | | + | | + | | + | | + | | + | | + | | + | | + | | + |
| 23. The discount rate(s) is stated. | - | Na | + | + | + | + | + | a)+  b)Na  c)Na | | + | | + | | + | | Na | | + | | + | | + | | Na | | + | | + | | Na | | Na | | + | | + | | + | | Na |
| 24. The choice of discount rate(s) is justified. | Na | Na | - | + | - | + | + | a)-  b)Na  c)Na | | - | | - | | - | | Na | | + | | - | | + | | Na | | + | | + | | Na | | Na | | + | | + | | - | | Na |
| 25. An explanation is given if costs and benefits are not discounted. | - | Na | Na | Na | Na | Na | Na | Na | | Na | | Na | | Na | | Na | | Na | | Na | | Na | | Na | | Na | | Na | | Na | | Na | | Na | | Na | | Na | | Na |
| 26. Details of statistical tests and confidence intervals are given for stochastic data. | Na | Na | Na | Na | Na | Na | Na | a) Na  b)Na  c)- | | Na | | Na | | - | | Na | | Na | | Na | | - | | Na | | Na | | Na | | Na | | Na | | Na | | Na | | Na | | Na |
| 27. The approach to sensitivity analysis is given. | + | + | + | + | + | + | + | + | | + | | + | | + | | + | | + | | + | | + | | + | | + | | + | | + | | + | | + | | + | | + | | + |
| 28. The choice of variables for sensitivity analysis is justified. | - | - | - | + | + | - | + | - | | - | | + | | - | | - | | - | | - | | - | | + | | + | | + | | - | | - | | - | | + | | - | | + |
| 29. The ranges over which the variables are varied are justified. | - | - | - | + | + | - | + | - | | + | | + | | - | | + | | - | | - | | - | | + | | + | | + | | - | | - | | - | | + | | - | | + |
| 30. Relevant alternatives are compared. | + | - | + | + | + | + | + | + | | - | | + | | + | | - | | - | | + | | - | | + | | + | | + | | - | | - | | + | | + | | + | | - |
| 31. Incremental analysis is reported. | + | + | + | + | + | + | + | + | | + | | + | | + | | + | | + | | + | | + | | + | | + | | + | | + | | - | | + | | + | | + | | + |
| 32. Major outcomes are presented in a disaggregated as well as aggregated form. | + | + | + | + | + | + | + | - | | + | | + | | + | | + | | + | | + | | + | | + | | + | | + | | + | | - | | + | | - | | + | | - |
| 33. The answer to the study question is given. | + | + | + | + | + | + | + | + | | + | | + | | + | | + | | + | | + | | + | | + | | + | | + | | + | | - | | + | | + | | + | | + |
| 34. Conclusions follow from the data reported. | + | + | + | + | + | + | + | + | | + | | + | | + | | + | | + | | + | | + | | + | | + | | + | | + | | - | | + | | + | | + | | + |
| 35. Conclusions are accompanied by the appropriate caveats. | + | + | + | + | + | + | + | + | | + | | + | | + | | + | | - | | + | | + | | + | | + | | + | | + | | + | | - | | + | | + | | + |
| **Philips´ checklist** | | | | | | | | | | | | | | | | | | | | | | | | | | | | | | | | | | | | | | | | |
| S1 Statement of decision problem/objective | | | | | | | | | | | | | | | | | | | | | | | | | | | | | | | | | | | | | | | | |
| 1. Is there a clear statement of the decision problem? | + | + | + | + | + | + | + | + | + | | + | | + | | + | | + | | + | | + | | + | | + | | + | | + | | + | | + | | + | | + | | + | |
| 2. Is the objective of the evaluation and model specified and consistent with the stated decision problem? | + | + | + | + | + | + | + | + | + | | + | | + | | + | | + | | + | | + | | + | | + | | + | | + | | + | | + | | + | | + | | + | |
| 3. Is the primary decision-maker specified? | - | - | - | - | - | - | - | - | - | | + | | - | | - | | - | | + | | - | | - | | - | | - | | + | | - | | - | | - | | - | | - | |
| S2 Statement of scope/perspective | | | | | | | | | | | | | | | | | | | | | | | | | | | | | | | | | | | | | | | | |
| 4. Is the perspective of the model stated clearly? | + | + | + | + | + | + | + | - | + | | + | | + | | - | | + | | + | | + | | + | | + | | + | | + | | + | | + | | + | | + | | + | |
| 5. Are the model inputs consistent with the stated perspective? | + | + | + | + | + | + | + | Na | - | | + | | + | | Na | | + | | + | | + | | + | | + | | + | | + | | + | | + | | + | | + | | + | |
| 6. Has the scope of the model been stated and justified? | + | + | + | + | + | + | + | + | + | | + | | + | | + | | + | | + | | + | | + | | + | | + | | + | | + | | + | | + | | + | | + | |
| 7. Are the outcomes of the model consistent with the perspective, scope and overall objective of the model? | + | + | + | + | + | + | + | + | - | | + | | + | | + | | + | | + | | + | | + | | + | | + | | + | | - | | + | | + | | + | | + | |
| S3 Rationale for structure | | | | | | | | | | | | | | | | | | | | | | | | | | | | | | | | | | | | | | | | |
| 8. Is the structure of the model consistent with a coherent theory of the health condition under evaluation? | - | - | - | + | + | + | + | - | + | | + | | + | | - | | - | | + | | - | | + | | + | | + | | - | | - | | - | | + | | + | | - | |
| 9. Are the sources of data used to develop the structure of the model specified? | - | + | - | + | + | + | + | - | - | | + | | - | | - | | - | | - | | - | | + | | + | | + | | + | | + | | + | | - | | - | | - | |
| 10. Are the causal relationships described by the model structure justified appropriately? | + | + | - | + | + | + | + | - | + | | + | | - | | + | | - | | + | | - | | + | | - | | - | | + | | + | | - | | + | | + | | + | |
| S4 Structural assumptions | | | | | | | | | | | | | | | | | | | | | | | | | | | | | | | | | | | | | | | | |
| 11. Are the structural assumptions transparent and justified? | + | - | - | + | + | + | + | - | + | | - | | - | | - | | - | | + | | - | | - | | - | | - | | - | | - | | - | | + | | + | | - | |
| 12. Are the structural assumptions reasonable given the overall objective, perspective and scope of the model? | + | - | - | + | + | + | + | - | + | | + | | - | | - | | - | | + | | - | | - | | - | | - | | - | | - | | - | | + | | + | | - | |
| S5 Strategies/comparators | | | | | | | | | | | | | | | | | | | | | | | | | | | | | | | | | | | | | | | | |
| 13. Is there a clear definition of the options under evaluation? | + | + | + | - | - | + | + | - | + | | - | | + | | + | | - | | - | | + | | + | | + | | + | | + | | + | | - | | + | | - | | + | |
| 14. Have all feasible and practical options been evaluated? | - | - | - | + | + | + | - | - | - | | + | | - | | - | | - | | - | | - | | - | | - | | - | | - | | - | | - | | - | | + | | - | |
| 15. Is there justification for the exclusion of feasible options? | - | - | - | Na | Na | Na | - | - | - | | Na | | - | | - | | - | | - | | - | | - | | - | | - | | + | | - | | - | | - | | Na | | - | |
| S6 Model type | | | | | | | | | | | | | | | | | | | | | | | | | | | | | | | | | | | | | | | | |
| 16. Is the chosen model type appropriate given the decision problem and specified causal relationships within the model? | - | - | + | + | + | + | + | a)+  b)-  c)- | - | | + | | + | | - | | + | | - | | + | | + | | + | | + | | - | | - | | + | | + | | + | | - | |
| S7 Time horizon | | | | | | | | | | | | | | | | | | | | | | | | | | | | | | | | | | | | | | | | |
| 17. Is the time horizon of the model sufficient to reflect all important differences between options? | - | - | - | - | - | + | - | a)+  b)-  c)- | - | | - | | + | | - | | - | | + | | - | | - | | - | | - | | - | | - | | - | | - | | - | | - | |
| 18. Are the time horizon of the model, the duration of treatment and the duration of treatment effect described and justified? | - | + | + | + | + | + | + | + | + | | + | | - | | + | | + | | + | | + | | + | | + | | + | | + | | + | | + | | + | | + | | + | |
| S8 Disease states/pathways | | | | | | | | | | | | | | | | | | | | | | | | | | | | | | | | | | | | | | | | |
| 19. Do the disease states (state transition model) or the pathways (decision tree model) reflect the underlying biological process of the disease in question and the impact of interventions? | + | + | - | + | + | + | + | - | + | | + | | + | | + | | + | | + | | + | | + | | + | | + | | - | | + | | + | | + | | + | | + | |
| S9 Cycle length | | | | | | | | | | | | | | | | | | | | | | | | | | | | | | | | | | | | | | | | |
| 20. Is the cycle length defined and justified in terms of the Ntural history of disease? | Na | Na | - | CD)+ UC)- | + | + | - | + | Na | | + | | + | | Na | | - | | Na | | + | | + | | - | | - | | Na | | Na | | + | | + | | - | | Na | |
| D1 Data identification | | | | | | | | | | | | | | | | | | | | | | | | | | | | | | | | | | | | | | | | |
| 21. Are the data identification methods transparent and appropriate given the objectives of the model? | + | - | - | + | + | + | + | - | + | | + | | - | | + | | - | | + | | - | | + | | - | | - | | - | | + | | - | | + | | + | | + | |
| 22. Where choices have been made between data sources, are these justified appropriately? | + | - | - | + | + | + | + | - | + | | + | | - | | - | | - | | Na | | Na | | + | | - | | - | | - | | + | | - | | + | | + | | + | |
| 23. Has particular attention been paid to identifying data for the important parameters in the model? | + | - | + | + | + | + | + | - | + | | + | | + | | + | | + | | + | | + | | + | | + | | + | | + | | + | | + | | + | | + | | + | |
| 24. Has the quality of the data been assessed appropriately? | - | - | + | + | + | - | + | - | - | | + | | - | | - | | - | | - | | - | | + | | - | | - | | - | | + | | - | | - | | + | | - | |
| 25. Where expert opinion has been used, are the methods described and justified? | Na | Na | - | + | + | Na | + | + | + | | - | | - | | Na | | + | | Na | | Na | | - | | - | | - | | + | | Na | | + | | + | | Na | | Na | |
| D2 Data modelling | | | | | | | | | | | | | | | | | | | | | | | | | | | | | | | | | | | | | | | | |
| 26. Is the data modelling methodology based on justifiable statistical and epidemiological techniques? | - | + | + | + | + | + | + | a)+  b)-  c)- | - | | + | | + | | - | | - | | - | | + | | + | | + | | + | | - | | - | | - | | - | | + | | - | |
| D2a Baseline data | | | | | | | | | | | | | | | | | | | | | | | | | | | | | | | | | | | | | | | | |
| 27. Is the choice of baseline data described and justified? | + | + | - | + | + | + | + | a)+  b)-  c)- | + | | + | | + | | + | | + | | + | | + | | + | | + | | + | | + | | + | | + | | + | | + | | + | |
| 28. Are transition probabilities calculated appropriately? | + | + | - | + | + | + | + | a)+  b)-  c)- | + | | + | | - | | + | | + | | + | | + | | + | | + | | + | | - | | + | | + | | + | | + | | + | |
| 29. Has a half-cycle correction been applied to both cost and outcome? | Na | Na | - | - | - | - | - | - | Na | | - | | - | | Na | | - | | Na | | - | | - | | - | | - | | Na | | Na | | - | | - | | - | | Na | |
| 30. If not, has this omission been justified? | Na | Na | - | - | - | - | - | - | Na | | - | | - | | Na | | - | | Na | | - | | - | | - | | - | | Na | | Na | | - | | - | | - | | Na | |
| D2b Treatment effects | | | | | | | | | | | | | | | | | | | | | | | | | | | | | | | | | | | | | | | | |
| 31. If relative treatment effects have been derived from trial data, have they been synthesised using appropriate techniques? | - | - | + | + | + | + | - | a)-  b)-  c)Na | + | | + | | Na | | - | | - | | - | | Na | | - | | - | | + | | - | | + | | - | | - | | + | | - | |
| 32. Have the methods and assumptions used to extrapolate short- term results to final outcomes been documented and justified? | - | - | - | - | - | + | + | - | - | | + | | - | | + | | - | | + | | + | | + | | - | | - | | Na | | - | | + | | + | | - | | Na | |
| 33. Have alternative assumptions been explored through sensitivity analysis? | - | - | - | - | - | - | + | - | - | | + | | - | | - | | - | | - | | - | | - | | - | | - | | Na | | - | | - | | + | | - | | Na | |
| 34. Have assumptions regarding the continuing effect of treatment once treatment is complete been documented and justified? | - | - | - | - | - | + | + | - | + | | Na | | - | | + | | + | | + | | - | | - | | - | | - | | + | | - | | + | | - | | - | | - | |
| 35. Have alternative assumptions been explored through sensitivity analysis? | - | - | - | - | - | - | + | - | - | | Na | | - | | - | | - | | - | | - | | - | | - | | - | | - | | - | | - | | - | | - | | - | |
| D2c Costs | | | | | | | | | | | | | | | | | | | | | | | | | | | | | | | | | | | | | | | | |
| 36. Are the costs incorporated into the model justified? | + | + | + | + | + | + | + | - | + | | + | | - | | + | | + | | + | | - | | + | | + | | - | | + | | + | | + | | + | | + | | + | |
| 37. Has the source for all costs been described? | + | + | + | + | + | + | + | - | + | | + | | - | | + | | + | | + | | - | | + | | + | | + | | + | | + | | + | | + | | + | | + | |
| 38. Have discount rates been described and justified given the target decision-maker? | - | Na | + | + | + | + | + | a)+  b)Na  c)Na | + | | + | | + | | Na | | + | | + | | + | | Na | | + | | + | | Na | | Na | | + | | + | | + | | Na | |
| D2d Quality of life weights (utilities) | | | | | | | | | | | | | | | | | | | | | | | | | | | | | | | | | | | | | | | | |
| 39. Are the utilities incorporated into the model appropriate? | + | - | - | - | - | + | + | - | - | | + | | - | | - | | + | | - | | + | | - | | + | | + | | - | | - | | + | | - | | - | | - | |
| 40. Is the source for the utility weights referenced? | + | + | + | + | + | + | + | + | + | | + | | + | | + | | + | | + | | + | | + | | + | | + | | + | | + | | + | | + | | + | | + | |
| 41. Are the methods of derivation for the utility weights justified? | + | + | + | + | + | + | + | + | + | | + | | + | | + | | + | | + | | + | | - | | + | | + | | + | | + | | + | | + | | + | | + | |
| D3 Data incorporation | | | | | | | | | | | | | | | | | | | | | | | | | | | | | | | | | | | | | | | | |
| 42. Have all data incorporated into the model been described and referenced in sufficient detail? | - | - | - | - | - | + | + | - | + | | + | | - | | - | | - | | - | | - | | - | | - | | - | | + | | - | | - | | - | | - | | - | |
| 43. Has the use of mutually inconsistent data been justified (i.e. are assumptions and choices appropriate)? | - | - | - | + | + | + | + | - | + | | - | | - | | - | | - | | - | | - | | - | | - | | - | | + | | - | | - | | + | | + | | - | |
| 44. Is the process of data incorporation transparent? | - | - | - | + | + | + | + | - | + | | + | | - | | + | | - | | - | | - | | + | | + | | + | | + | | + | | - | | + | | + | | + | |
| 45. If data have been incorporated as distributions, has the choice of distribution for each parameter been described and justified? | - | - | - | + | + | - | + | Na | - | | + | | Na | | Na | | Na | | + | | + | | + | | + | | + | | - | | + | | Na | | + | | + | | + | |
| 46. If data have been incorporated as distributions, is it clear that second order uncertainty is reflected? | - | - | - | + | + | - | - | Na | + | | + | | Na | | Na | | Na | | - | | + | | - | | - | | - | | - | | + | | Na | | - | | + | | - | |
| D4 Assessment of uncertainty | | | | | | | | | | | | | | | | | | | | | | | | | | | | | | | | | | | | | | | | |
| 47. Have the four principal types of uncertainty been addressed? | - | - | - | + | + | - | + | - | - | | - | | - | | - | | + | | - | | - | | - | | + | | + | | - | | - | | - | | - | | - | | - | |
| 48. If not, has the omission of particular forms of uncertainty been justified? | - | - | - | Na | Na | - | Na | - | - | | - | | - | | - | | Na | | - | | - | | - | | Na | | Na | | - | | - | | - | | - | | - | | - | |
| D4a Methodological | | | | | | | | | | | | | | | | | | | | | | | | | | | | | | | | | | | | | | | | |
| 49. Have methodological uncertainties been addressed by running alternative versions of the model with different methodological assumptions? | - | - | - | + | + | + | + | - | + | | + | | - | | - | | + | | + | | + | | - | | + | | + | | - | | - | | + | | + | | + | | - | |
| D4b Structural | | | | | | | | | | | | | | | | | | | | | | | | | | | | | | | | | | | | | | | | |
| 50. Is there evidence that structural uncertainties have been addressed via sensitivity analysis? | + | + | + | + | + | + | + | + | + | | + | | + | | + | | + | | + | | + | | + | | + | | + | | + | | + | | + | | + | | + | | + | |
| D4c Heterogeneity | | | | | | | | | | | | | | | | | | | | | | | | | | | | | | | | | | | | | | | | |
| 51. Has heterogeneity been dealt with by running the model separately for different subgroups? | - | - | - | + | + | - | + | - | - | | - | | - | | - | | + | | - | | - | | - | | + | | + | | - | | - | | + | | - | | - | | - | |
| D4d Parameter Attributes of good practice | | | | | | | | | | | | | | | | | | | | | | | | | | | | | | | | | | | | | | | | |
| 52. Are the methods of assessment of parameter uncertainty appropriate? | - | - | - | + | + | + | + | - | + | | + | | - | | + | | + | | + | | + | | + | | + | | + | | + | | + | | + | | + | | + | | + | |
| 53. If data are incorporated as point estimates, are the ranges used for sensitivity analysis stated clearly and justified? | - | - | - | + | + | - | + | - | + | | + | | - | | + | | - | | - | | - | | + | | - | | - | | - | | - | | - | | + | | - | | + | |
| C1 Internal consistency | | | | | | | | | | | | | | | | | | | | | | | | | | | | | | | | | | | | | | | | |
| 54. Is there evidence that the mathematical logic of the model has been tested thoroughly before use? | - | + | - | + | + | + | + | - | - | | - | | - | | - | | - | | - | | - | | + | | + | | - | | - | | + | | + | | - | | - | | - | |
| C2 External consistency | | | | | | | | | | | | | | | | | | | | | | | | | | | | | | | | | | | | | | | | |
| 55. Are any counterintuitive results from the model explained and justified? | + | Na | Na | + | + | + | + | Na | Na | | Na | | - | | Na | | + | | Na | | Na | | + | | Na | | Na | | + | | + | | Na | | + | | + | | Na | |
| 56. If the model has been calibrated against independent data, have any differences been explained and justified? | Na | Na | Na | Na | Na | Na | Na | Na | Na | | Na | | Na | | Na | | Na | | Na | | Na | | Na | | Na | | Na | | Na | | Na | | Na | | Na | | Na | | Na | |
| 57. Have the results of the model been compared with those of previous models and any differences in results explained? | + | - | - | + | + | + | + | + | - | | + | | + | | + | | + | | + | | + | | + | | - | | - | | + | | + | | + | | + | | + | | - | |
| **Consolidated health Economic Evaluation Reporting Standards (CHEERS) guideline** | | | | | | | | | | | | | | | | | | | | | | | | | | | | | | | | | | | | | | | | |
| Title and abstract | | | | | | | | | | | | | | | | | | | | | | | | | | | | | | | | | | | | | | | | |
| 1. Title | - | - | - | + | + | + | - | + | | - | | + | | - | | + | | + | | + | | + | | + | | + | | + | | + | | + | | + | | + | | + | | + |
| 2. Abstract | + | + | + | + | + | + | + | - | | + | | + | | + | | + | | + | | + | | + | | + | | + | | + | | + | | + | | + | | + | | + | | + |
| 3. Background and objectives | + | + | + | + | + | + | + | + | | + | | + | | + | | + | | + | | + | | + | | + | | + | | + | | + | | + | | + | | + | | + | | + |
| Methods | | | | | | | | | | | | | | | | | | | | | | | | | | | | | | | | | | | | | | | | |
| 4. Target population and subgroups | + | + | + | + | + | + | + | + | | + | | + | | + | | + | | + | | - | | + | | + | | + | | + | | + | | + | | - | | + | | + | | + |
| 5. Setting and location | + | + | + | + | + | + | + | + | | + | | + | | + | | + | | + | | + | | + | | + | | + | | + | | + | | + | | + | | + | | + | | + |
| 6. Study perspective | + | + | + | + | + | + | + | - | | + | | + | | + | | - | | + | | + | | + | | + | | + | | + | | + | | + | | + | | + | | + | | + |
| 7. Comparators | + | - | + | + | + | + | + | + | | - | | + | | + | | + | | + | | + | | + | | + | | + | | + | | - | | + | | + | | + | | + | | + |
| 8. Time horizon | + | + | + | + | + | + | + | a)+  b)-  c)- | | + | | + | | + | | + | | + | | + | | + | | + | | + | | + | | + | | + | | + | | + | | + | | + |
| 9. Discount rate | - | Na | + | + | + | + | + | a)+  b)Na  c)Na | | + | | + | | + | | Na | | + | | + | | + | | Na | | + | | + | | Na | | Na | | + | | + | | + | | Na |
| 10. Choice of health outcomes | + | + | + | + | + | + | + | + | | + | | + | | + | | + | | + | | + | | + | | + | | + | | + | | + | | + | | + | | + | | + | | + |
| 11a. Measurement of effectiveness (Single study-based estimates) | Na | Na | Na | Na | Na | Na | Na | a)Na  b)Na  c)- | | Na | | Na | | - | | Na | | Na | | Na | | - | | Na | | Na | | Na | | Na | | Na | | Na | | Na | | Na | | Na |
| 11b. Measurement of effectiveness (Synthesis-based estimates) | - | - | - | + | + | + | + | a)-  b)-  c)Na | | + | | + | | Na | | + | | - | | - | | Na | | + | | - | | + | | - | | + | | - | | - | | + | | + |
| 12. Measurement and valuation of preference based outcomes | + | + | + | + | + | + | + | + | | + | | + | | + | | + | | + | | + | | + | | + | | + | | + | | + | | + | | + | | + | | + | | + |
| 13a. Estimating resources and costs (Single study-based economic evaluation) | Na | Na | Na | Na | Na | Na | Na | Na | | Na | | Na | | Na | | Na | | Na | | Na | | Na | | Na | | Na | | Na | | Na | | Na | | Na | | Na | | Na | | Na |
| 13b. Estimating resources and costs (Model-based economic evaluation) | + | + | + | + | + | + | + | - | | + | | + | | + | | + | | + | | + | | - | | + | | + | | + | | + | | + | | + | | - | | + | | + |
| 14. Currency, price date, and conversion | + | + | + | CD)+  UC)- | - | + | + | - | | + | | + | | - | | + | | - | | + | | - | | + | | + | | + | | + | | + | | - | | + | | + | | + |
| 15. Choice of model | + | + | + | + | + | + | + | - | | + | | + | | + | | + | | + | | - | | + | | - | | + | | + | | + | | + | | + | | + | | + | | - |
| 16. Assumptions | + | - | - | + | + | + | + | - | | + | | - | | - | | - | | - | | - | | - | | - | | - | | - | | + | | - | | - | | - | | + | | - |
| 17. Analytical methods | - | - | - | - | - | - | + | - | | + | | + | | - | | - | | - | | - | | - | | + | | - | | - | | - | | - | | - | | + | | - | | - |
| Results | | | | | | | | | | | | | | | | | | | | | | | | | | | | | | | | | | | | | | | | |
| 18. Study parameters | + | - | + | + | - | - | + | - | | + | | + | | - | | - | | - | | - | | - | | + | | - | | - | | - | | + | | - | | + | | + | | + |
| 19. Incremental costs and outcomes | + | + | + | + | + | + | + | - | | + | | + | | + | | + | | + | | + | | + | | + | | + | | + | | + | | - | | + | | + | | + | | - |
| 20a. Characterising uncertainty (Single study-based economic evaluation) | Na | Na | Na | Na | Na | Na | Na | Na | | Na | | Na | | Na | | Na | | Na | | Na | | Na | | Na | | Na | | Na | | Na | | Na | | Na | | Na | | Na | | Na |
| 20b. Characterising uncertinty (Model-based economic evaluation) | - | - | - | + | + | - | + | - | | + | | + | | - | | - | | + | | + | | + | | + | | + | | + | | - | | + | | + | | + | | + | | + |
| 21. Characterising heterogenity | - | - | - | + | + | - | + | - | | - | | - | | - | | - | | + | | - | | - | | - | | + | | + | | - | | - | | + | | - | | - | | - |
| Discussion | | | | | | | | | | | | | | | | | | | | | | | | | | | | | | | | | | | | | | | | |
| 22. Study findings, limitations, generalisability, and current knowledge | + | + | + | + | + | + | + | + | | + | | + | | + | | + | | - | | + | | + | | + | | + | | + | | + | | + | | - | | + | | + | | + |
| Others | | | | | | | | | | | | | | | | | | | | | | | | | | | | | | | | | | | | | | | | |
| 23. Source of funding | + | - | + | + | + | + | + | + | | + | | + | | - | | + | | + | | + | | - | | + | | + | | + | | + | | - | | + | | + | | + | | + |
| 24. Conflicts of interest | + | + | + | + | + | + | + | + | | + | | + | | - | | + | | + | | + | | + | | + | | + | | - | | - | | - | | + | | + | | + | | + |
| +, Fulfillment of item; −, No fulfillment of item; Na, Not applicable. | | | | | | | | | | | | | | | | | | | | | | | | | | | | | | | | | | | | | | | | |
