# Supplementary material for: A Systematic Review of the Cost-Effectiveness of Biologics for the Treatment of Inflammatory Bowel Diseases
Source: PLoS One. 2015 Dec 16;10(12):e0145087. doi: 10.1371/journal.pone.0145087 (PMC4682717; doi:10.1371/journal.pone.0145087)
Supplement: S3 Table — (DOC) [file pone.0145087.s005.doc]

| **Section/topic** | **#** | **Checklist item** | | **Reported on page #** |
| --- | --- | --- | --- | --- |
| **TITLE** | | | |  |
| Title | 1 | A Systematic Review of the Cost-effectiveness of Biologics for the Treatment of Inflammatory Bowel Diseases | | 1 |
| **ABSTRACT** | | | |  |
| Structured summary | 2 | *Background:* Despite the high cost of biologics used for the treatment of inflammatory bowel diseases, Crohn’s disease and ulcerative colitis are refractory to conventional treatment. In order to allocate healthcare spending efficiently, biologics for inflammatory bowel diseases are an important target for cost-effectiveness analyses. The aim of this study was to systemically review all published literature on the cost-effectiveness of biologics for inflammatory bowel diseases and to evaluate the methodological quality of cost-effectiveness analyses.  *Methods:* A literature search was performed using Medline (Ovid), Cochrane Library, and SCOPUS. All cost-utility analyses comparing biologics with conventional medical treatment, another biologic treatment, placebo, or surgery for the treatment of inflammatory bowel diseases in adults were included in this review. All costs were converted to the 2014 euro. The methodological quality of the included studies was assessed by Drummond’s, Philips‘, and the Consolidated Health Economic Evaluation Reporting Standards checklist.  *Results:* Altogether, 25 studies were included in the review. Among the patients refractory to conventional medical treatment, the incremental cost-effectiveness ratio ranged from dominance to 549,335 €/Quality-Adjusted Life Year compared to the incremental cost-effectiveness ratio associated with conventional medical treatment. When comparing biologics with another biologic treatment, the incremental cost-effectiveness ratio ranged from dominance to 24,012,483 €/Quality-Adjusted Life Year. A study including both direct and indirect costs produced more favorable incremental cost-effectiveness ratios than those produced by studies including only direct costs.  *Conclusions:* With a threshold of 35,000 €/Quality-Adjusted Life Year, biologics are cost-effective for the treatment of active and severe inflammatory bowel disease. Between biologics, the cost-effectiveness remains unclear. | | 2 |
| **INTRODUCTION** | | | |  |
| Rationale | 3 | Crohn’s disease (CD) and ulcerative colitis (UC) are the principal types of inflammatory bowel diseases (IBDs) [1,2]. IBDs, which are chronic diseases, are characterized by inflammation of the mucosal lining of the gastrointestinal tract. Their worldwide incidence has increased during the last decade, but the annual incidence and prevalence of CD and UC are the highest in Northern Europe and in North America [3]. The incidence of CD is 12.7 per 100,000 person-years in Europe and 20.2 person-years in North America, while the incidence of UC is 24.3 in Europe and 19.2 in North America. Unemployment, sick leave, and permanent work disability are more commonly associated with patients with IBD than with the general population [4]. IBDs affect mainly young adults, causing an even greater economic burden.  Treatment of IBDs is aimed at relieving the symptoms and complications of IBDs as well as preventing recurrence and improving the patient’s quality of life [5,6]. IBD patients usually require lifelong medical treatment. Both CD and UC are treated with conventional medical treatment comprising corticosteroids, aminosalicylates, and immunomodulators (e.g., azathioprine and 6-mercaptopurine). Other treatment options include surgery and diet therapy [5–7]. Biologic drugs based on two different mechanisms of action are currently available for the treatment of IBDs [8]. Infliximab (IFX), adalimumab (ADA), golimumab, and certolizumab pegol (CTZ) are tumor necrosis factor (TNF) inhibitors while natalizumab (NTZ) and vedolizumab target the cell adhesion molecule α4-integrin [9,10]. Biologics are used to treat IBD refractory to corticosteroids or immunomodulators or IBD patients who are steroid-dependent or steroid-intolerant [5,6]. However, biologics are significantly more expensive than conventional drugs [11,12]. They have been shown to be effective in inducing and maintaining remission of IBD [5,6,8,13–15]. The evidence on the cost-effectiveness of biologics for the treatment of IBD is limited, and the results of previous systematic reviews are inconsistent and incomplete [16–20]. | | 3 |
| Objectives | 4 | The aim of this systematic review is to evaluate existing relevant evidence regarding the cost-effectiveness of biologics for the treatment of IBDs. The cost-effectiveness of biologics is compared with placebo treatment, conventional medical treatment, surgery, and another biologic treatment for adults with diagnosed IBD. The aim of this review is also to analyze the source of effectiveness of CEAs. Furthermore, this review assesses the quality of the included CEAs using three different quality assessment checklists. | | 4 |
| **METHODS** | | | |  |
| Protocol and registration | 5 | - | | Not applicable |
| Eligibility criteria | 6 | The study selection was based on the inclusion and the exclusion criteria formulated by the framework of PICOTS i.e., population, intervention, comparator, outcome, timing, and setting (S2 Table) [25]. | | 5, S2 Table |
| Information sources | 7 | A comprehensive literature search on the cost-effectiveness of biologics for the treatment of IBDs was performed using Medline (Ovid), Cochrane Library (Cochrane Database of Systematic Reviews, Database of Abstracts of Reviews of Effects, Cochrane Central Register of Controlled Trials, Cochrane Methodology Register, Heath Technology Assessment Database, and NHS Economic Evaluation Database), and SCOPUS (including Embase) in June 2014. The search strategies were developed together with an information specialist. The reference lists of relevant articles were scrutinized. Furthermore, the grey literature and other relevant websites and databases (Centre for Reviews and Dissemination, Current Controlled Trials, Clinical Trials.gov, and PROSPERO) were hand-searched for relevant studies. | | 4 |
| Search | 8 | The electronic search strategy was based on patients (IBD, CD, or UC), intervention (biologics), and outcomes (ICER) in different spellings (S1 File). The biologics granted a marketing authorization by the European Medicines Agency (EMA) or US Food and Drug Administration (FDA) before May 2014 were included in the literature search strategy [9,10]. No restriction was set based on the year of the publication. | | 5, S1 File |
| Study selection | 9 | The study selection procedure encompassed three main stages. At the first stage, hits from the electronic databases were imported into reference management software (RefWorks). After removing duplicate citations, the second stage focused on the evaluation of the remaining studies based on their titles and abstracts. Studies clearly indicated as irrelevant to the study subject were excluded. The full articles retrieved that met the inclusion criteria are included in the current review. The identified abstracts and full texts were screened for eligibility by one reviewer (SH) and the second reviewer (MB) was consulted. | | 5 |
| Data collection process | 10 | Our data extraction form was based on the Cochrane Handbook for Systematic Reviews of Intervention and the abstract form of the NHS Economic Evaluation Database [26,27].  Data were extracted using Microsoft Excel and performed by one assessor (SH) and ambiguities were solved by another assessor (MB) for accuracy. | | 5 |
| Data items | 11 | The following items were extracted: patients, interventions, controls, study design (the type of economic evaluation and modeling, perspective, time horizon, country, included costs, the methods of measuring and valuing outcomes and benefits, discount rate, currency, price year, and the type of sensitivity analysis) and outcomes (total costs and benefits, ICER, and the results of sensitivity analysis). | | 5 |
| Risk of bias in individual studies | 12 | The methodological quality of the studies was assessed using three standardized checklists. All studies were assessed using Drummond’s checklist, published by the British Medical Journal Working Party, and the Consolidated Health Economic Evaluation Reporting Standards (CHEERS) guidelines [30,31]. In addition, economic evaluations using modeling methods were assessed using Philips’ checklist [32]. The quality assessment was conducted by one assessor (SH) and ambiguities were resolved by consulting another assessor (MB). | | 6 |
| Summary measures | 13 | ICERs were presented as principal outcomes. The cost-effectiveness of biologics was analyzed using the willingness-to-pay threshold of €35,000/QALY. | | 6 |
| Synthesis of results | 14 | The results of the included CUAs were stratified into 4 subgroups by the type of previous treatments: 1) the cost-effectiveness of biologics in patients without previous treatment, 2) the cost-effectiveness of biologics in patients with previous conventional medical treatment, 3) the cost-effectiveness of biologics in patients with previous surgery, and 4) the cost-effectiveness of biologics in patients with previous biologic treatment. Biologic treatments were stratified under three dosing regimens: a single dose, an episodic treatment, or a maintenance treatment. ICERs were presented as principal outcomes. The cost-effectiveness of biologics was analyzed using the willingness-to-pay threshold of 35,000 €/QALY. A quantitative synthesis of the study results was not possible because of heterogeneous study designs. | | 6 |
| Risk of bias across studies | 15 | | - | - |
| Additional analyses | 16 | | - | Not applicable |
| **RESULTS** | | | |  |
| Study selection | 17 | | The database search identified 1828 references, of which 461 were removed as duplicates, leaving 50 studies to be screened by abstracts and titles for further evaluation. After the assessment of the full text, 31 studies were excluded (S3 File) and 19 studies were included in the review. Additionally, six full-text articles were included, of which two were found from the bibliographies of already included studies [33,34] and four from the structured abstracts identified by the literature search [17,20,35,36]. The hand search revealed no further publications. Altogether, 25 studies were included in the review [17,19,20,33–54]. Study selection is presented in a flow diagram in Fig. 1. | 6, 7, Fig 1, S3 File |
| Study characteristics | 18 | | All CUAs involved economic evaluation modeling, of which 17 and 7 were focused on CD and UC, respectively, while one study featured both diagnoses. IFX, ADA, NTZ, and CTZ were studied in 22, 8, two, and one CUAs, respectively. All studies were conducted in North America or in Europe. One study considered both direct and indirect costs [47]. The study designs, the interventions, and the comparators of the CUAs were heterogeneous. Table 1 presents characteristics of the studies. | 7, Table 1 |
| Risk of bias within studies | 19 | | The mean amount of fulfilled criteria were 24.9 out of 35 (median 26, range 14–30), 29.6 out of 57 (median 29, range 14–46), and 18.2 out of 24 (median 18, range 10–23) for Drummond’s checklist, Philip’s checklist, and the CHEERS guideline, respectively. Studies by Assasi et al, Bryan et al, and Dretzke et al, which all are Health Technology Assessment (HTA) reports, fulfilled most criteria of the applicable items [19,20,35]. The quality elements most commonly omitted from the economic analyses were information on adjustments for data identification, baseline data, treatment effects, data incorporation, and assessment of uncertainty (S4 Table). | 22, Fig 2, S4 Table |
| Results of individual studies | 20 | | *Cost-Effectiveness of Biologics in Patients with No Previous Treatment*  In two studies, the cost-effectiveness of biologics was evaluated in CD patients with no previous treatment (Table 2) [39,48]. In comparison with conventional drugs for the treatment of fistulizing CD, ICERs ascended in excess of 400,000 €/QALY [39] while for newly diagnosed luminal CD IFX was dominant [48]. No CEAs of biologics in UC patients without earlier treatment were found (Table 3).  *Cost-Effectiveness of Biologics in Patients with Previous Conventional Medical Treatment*  The cost-effectiveness of biologics in CD patients with previous conventional medical treatment was investigated in 12 studies (Table 2) [17,19,20,40–42,44,46,47,49,50,54]. For CD, ICERs for the biologics ranged from dominance to 549,335 €/QALY when compared with those of conventional medical treatment [17,19,20,40,41,46,47]. ADA as an intervention treatment resulted in more frequently lower ICERs than did IFX in comparison with conventional medical treatment [19,20,40,41]. IFX in comparison with surgery was not found to be cost-effective, with ICERs in excess of 77,000 €/QALY [44]. Between biologics cost-effectiveness was investigated in four studies [20,40,50,54]. ICERs above 300,000 €/QALY were seen when comparing IFX with ADA [20,40], while ADA maintenance treatment appeared to be dominant in comparison with IFX maintenance treatment [54].  Two studies evaluated the cost-effectiveness of biologics for different activity levels of CD resulting in more favorable ICERs for severe CD than for moderate CD [19,47]. The cost-effectiveness of biologics for fistulizing CD was examined in two studies (ICERs above 51,000 €/QALY) [42,46] and for luminal CD in two studies (ICERs above 45,000 €/QALY) [44,46]. Biologic induction treatment resulted in lower ICERs than maintenance treatment [19]. In one study, IFX and corticosteroid combination treatment was shown to be cost-effective in comparison with IFX monotherapy [49]. One study found more favorable ICER when including both direct and indirect costs than only direct costs [47].  Eight CUAs evaluated the cost-effectiveness of biologics in UC patients with previous conventional medical treatment (Table 3) [20,33–36,51–53]. ICER remained below 35,000 €/QALY when comparing IFX with either conventional medical treatment, surgery, or placebo treatment for UC patients with acute exacerbation requiring hospitalization [33–35]. When investigating the cost-effectiveness of IFX for patients with moderate-to-severe UC, ICER ranged from 33,067 €/QALY to 407,499 €/QALY [20,36,51–53].  *Cost-Effectiveness of Biologics in Patients with Previous Surgery*  The cost-effectiveness of biologics in CD patients having undergone intestinal resection was investigated in two CUAs (Table 2) [37,43]. IFX in comparison with conventional medical treatment was not cost-effective, producing extremely unfavorable ICERs above 1,400,000 €/QALY. No studies investigated the cost-effectiveness of biologics in UC patients with previous surgery (Table 3).  *Cost-Effectiveness of Biologics in Patients with Previous Biologic Treatment*  The cost-effectiveness of biologics in CD patients with prior biologic treatment was investigated in two CUAs (Table 2) [38,45]. Neither IFX dose escalation in comparison with second-line ADA nor third-line CTZ in comparison with NTZ was cost-effective (ICERs above 300,000 €/QALY). No studies evaluated the cost-effectiveness of biologics in UC patients with prior TNF inhibitor treatment (Table 3).  *Effectiveness Data*  In all studies, the source of effectiveness was based on at least one randomized controlled trial (RCT). One study used real life data published by specialized inflammatory bowel disease clinics and compared those findings with data from RCTs [52].  In 13 studies focused on CD, utility values were obtained by the Standard Gamble (SG) valuation technique [17,20,38–40,42–45,47,49,50,54]. In twelve studies, the utilities were derived from study by Gregor et al [56] which used the SG method in CD patients to define utility scores and correlated them with the Crohn’s disease Activity Index (CDAI) [17,20,38,40,42–45,47,49,50,54]. In two studies [37,46], health state preferences were driven from the study by Casellas et al [55] which estimated health state preferences of Spanish CD patients using the European Quality of Life Instrument 5 D (EQ-5D) and converted to utilities using UK tariffs. In two studies [41,48] the estimated EQ-5D utility scores were converted from CDAI scores based on the algorithm developed by Buxton et al [57].  In three studies concerning UC [33,34,51], the utility scores were obtained from an UC patient survey carried out in Cardiff Hospital using the EQ-5D and valued using UK tariffs [58]. Utilities were further classified into health states by a Simple Clinical Colitis Activity Index (SCAI). Two studies [20,53] used utilities from patients using Time Trade-off (TTO) valuation technique [59]. | 14-22, Tables 2 and 3 |
| Synthesis of results | 21 | | - | Not applicable |
| Risk of bias across studies | 22 | | - | - |
| Additional analysis | 23 | | - | Not applicable |
| **DISCUSSION** | | | |  |
| Summary of evidence | 24 | | Altogether, 25 studies were included in this systematic review. The number of the included studies in this review was higher than in previously published reviews for IBD [16–20]. However, it is noteworthy that articles by Blackhouse et al and Xie et al are part of the study by Assasi et al [20,40,53]. A majority of the included studies used IFX or ADA as an intervention treatment, while NTZ and CTZ were investigated only in few studies, and none of the studies considered golimumab. Because of the variability in data input and heterogeneous study designs, the quantitative synthesis of the studies was not possible.  On the basis of the current review and willingness-to-pay threshold of 35,000 €/QALY, biologics in comparison with conventional medical treatment and placebo treatment were found to be cost-effective for severe CD in remission induction, while for maintenance treatment cost-effectiveness remained unclear. Biologics were not cost-effective in comparison with surgery for the treatment of severe CD. In moderate CD, biologics did not seem to be cost-effective. Biologics were found not to be cost-effective among CD patients having undergone intestinal resection. ADA was shown to be a more cost-effective biologic treatment option than IFX. Cost-effectiveness between individual biologics remained unclear, however.  Biologics were cost-effective for the treatment of acute exacerbation of severely active UC when compared with either conventional medical treatment, surgery, or placebo treatment. For moderate UC, biologics were not cost-effective. The cost-effectiveness between different biologics remained unclear in UC. | 22,23 |
| Limitations | 25 | | Most studies used the perspective of the local public health care service or the insurance system while only paying attention to direct costs. Only one study included both direct and indirect costs and reported more favorable ICER when considering both direct and indirect costs in comparison with only direct costs [47]. No clear guidelines exist on how productivity losses should be determined causing concern for the validity of the cost estimates. Included cost components and their valuing methods can be difficult to identify based on the publications. Furthermore, productivity costs included in CUA may cause a risk of double-counting as the impact of morbidity is already included in the calculation of QALY [24]. IBDs as chronic diseases are usually diagnosed in early adulthood causing a severe impact on productivity costs. Consequently, it is appropriate to include indirect costs in CUA, but indirect costs should be presented separately from direct costs [22,24].  When evaluating effectiveness, it is scientifically and ethically important to use the most appropriate alternative treatment as the control group. The comparator with a good efficacy and safety profile should act as the most cost-effective alternative treatment and is usually the intervention most used in clinical practice [30,65]. However, recommendations on the appropriate comparator vary across countries and depend on the research question [65]. A majority of the included studies used the “standard care” or “usual care” as the comparator.  Source of effectiveness data has substantial influence on model results. RCT data was used as effectiveness data in all included CUAs. RCTs give information about efficacy determined in ideal circumstances and cause a risk of overestimating effectiveness in comparison with the treatment in routine healthcare. Therefore, it is plausible to assume that the CUAs using RCTs as a source of effectiveness produce lower ICERs than real-world data. Contrary to that assumption, only one of the included studies derived information from real life studies and resulted in more favorable ICERs when using response rates from real life data rather than from RCTs [52]. However, the uncertainty in economic evaluations, especially in modeling studies, can arise from numerous methodological disagreements among analyses. Uncertainty caused by e.g., using multiple data sources and extrapolation beyond the time horizon of the study involving the use of assumptions was tested by sensitivity analysis in most studies. The correlation between the length of the time horizon and cost-effectiveness remains unclear.  In most studies, the source of utility data was reported inadequate and considerable variation existed in the instruments used to collect it. Direct elicitation methods (e.g., SG, TTO) were used more frequently than indirect methods (e.g., EQ-5D). With direct methods patients directly score their preferences for health states and make judgments based on their own relative values, while indirect methods are based on the patients’ responses to surveys about various aspects of health states [23]. The methods of direct elicitation can be complex and time consuming. In most cases, indirect utility estimates were obtained by determining the relationship between values on a disease-specific measure to a generic quality of life measure. This is necessary because of the fact that the generic measures have been applied in few studies, while disease specific measures such as CDAI are commonly used in RCTs. The application of different algorithms for conversions creates a further source of heterogeneity in ICER estimates.  When considering the earlier published systematic reviews, only one study used the standardized quality assessment checklist to evaluate the quality of the included CUAs. As far as we know, this is the first systematic review assessing the quality of economic evaluations by three different checklists. Drummond’s checklist is recommended to inform appraisal of the methodological quality of full economic evaluations [30,66]. Drummond’s checklist is relevant but not sufficient for modeling studies. Therefore, the modeling’s were also assessed using Philip’s checklist [30,32,66]. The CHEERS guideline includes additional items relating to the author’s disclosure of funding sources and conflicts of interest, sufficient information in article titles, and structured abstracts [31]. The CHEERS guideline evaluates the reporting of the study while Drummond’s checklist and Philips’ checklist are designed to assess the methodological quality of economic evaluations.  The amount of the fulfilled items according to Drummond’s checklist and the CHEERS guideline was higher than using Philips’ checklist. The reasons may be aims of the checklist and the extensiveness of Philips’ checklist including several topics relevant to modeling studies and not considered in Drummond’s checklist and the CHEERS guideline. On average, the same CUAs fulfilled the highest amount of the applicable items according to all three checklists. Most of the studies, which fulfilled most criteria of quality assessment checklists, were HTA reports. Almost half of the included CUAs were funded by the pharmaceutical company or authors had received funding from the pharmaceutical companies during the research project [33,34,40,43,45–47,51,53,54]. Many of the studies funded by the pharmaceutical company produced favorable ICERs [33,34,46,47,51,54]. However, it remained unclear whether the source of funding had an effect on the study results. In addition, the relation between the studies funded by a pharmaceutical company and fulfillment of applicable quality assessment criteria was found to be unclear.  The current review was carefully designed beforehand and documented transparently, improving the validity of the study. The study selection, the data extraction, and the quality assessment were performed by one assessor and any ambiguity was resolved with a second assessor to avoid human mistakes and to improve the reliability of the study. The comprehensive literature search was utilized to minimize bias. The intervention treatments included in the search strategy were limited to biologics that had been granted a marketing authorization by the EMA or FDA for the treatment of IBD. Vedolizumab was not included in the search strategy because its marketing authorization was not granted until the planning and realization of the search strategy was completed.  However, because of a limited amount of available CEAs and some inconsistent results, conclusions remain partially uncertain. Furthermore, variability in data input and heterogeneity in study designs made it challenging to compare studies reliably. To improve the reporting of an individual CEA, it is appropriate to use quality assessment checklists. When using checklists, economic evaluations become more consistent, transparent, and informative. | 24-26 |
| Conclusions | 26 | | In conclusion, biologics were found to be cost-effective for the induction treatment of active and severe IBD. Biologics were not shown to be cost-effective for maintenance treatment. Between biologics the cost-effectiveness remains unclear. | 27 |
| **FUNDING** | | | |  |
| Funding | 27 | | None declared | Submission |

*From:*  Moher D, Liberati A, Tetzlaff J, Altman DG, The PRISMA Group (2009). Preferred Reporting Items for Systematic Reviews and Meta-Analyses: The PRISMA Statement. PLoS Med 6(6): e1000097. doi:10.1371/journal.pmed1000097

For more information, visit: **www.prisma-statement.org**.
